# Early intervention anti-Aβ immunotherapy attenuates microglial activation without inducing exhaustion at residual plaques

**DOI:** 10.1186/s13024-025-00878-1

**Published:** 2025-08-20

**Authors:** Lis de Weerd, Selina Hummel, Stephan A. Müller, Iñaki Paris, Thomas Sandmann, Marie Eichholtz, Robin Gröger, Amelie L. Englert, Stephan Wagner, Connie Ha, Sonnet S. Davis, Valerie Warkins, Dan Xia, Brigitte Nuscher, Anna Berghofer, Marvin Reich, Astrid F. Feiten, Kai Schlepckow, Michael Willem, Stefan F. Lichtenthaler, Joseph W. Lewcock, Kathryn M. Monroe, Matthias Brendel, Christian Haass

**Affiliations:** 1 German Center for Neurodegenerative Diseases (DZNE), Munich, Germany; 2 Department of Nuclear Medicine, University Hospital of Munich, Ludwig- Maximilians University (LMU), Munich, Germany; 3 Denali Therapeutics, Inc, South San Francisco, USA; 4 Metabolic Biochemistry, Biomedical Center, Faculty of Medicine, Ludwig- Maximilians University (LMU), Munich, Germany; 5 Munich Cluster for Systems Neurology (SyNergy), Munich, Germany; 6 Biochemistry Master’s Program, Gene Center Munich, Ludwig-Maximilians University (LMU), Munich, Germany; 7 Neuroproteomics, School of Medicine and Health, Klinikum Rechts der Isar, Technical University of Munich (TUM), Munich, Germany; 8 Present Address: Department of Neurology and Neurological Sciences, Stanford University School of Medicine, Stanford University, Stanford, CA USA

**Keywords:** Alzheimer’s disease (AD), Immunotherapy, Aducanumab, Amyloid plaque, Microglia, Trem2

## Abstract

Anti-amyloid β-peptide (Aβ) immunotherapy was developed to reduce amyloid plaque pathology and slow cognitive decline during progression of Alzheimer’s disease. Efficient amyloid clearance has been proven in clinical trials testing anti-Aβ antibodies, by their impact on cognitive endpoints correlating with the extent of amyloid removal. However, treatment is associated with adverse side effects, such as oedema and haemorrhages, which are potentially linked to the induced immune response. To improve the safety profile of these molecules, it is imperative to understand the consequences of anti-Aβ antibody treatment on immune cell function. Here, we investigated the effects of long-term chronic anti-Aβ treatment on amyloid plaque pathology and microglial response in the APP-SAA triple knock-in mouse model with an intervention paradigm early during amyloidogenesis. Long-term treatment with anti-Aβ results in a robust and dose-dependent lowering of amyloid plaque pathology, with a higher efficiency for reducing diffuse over dense-core plaque deposition. Analysis of the CSF proteome indicates a reduction of markers for neurodegeneration including Tau and α-Synuclein, as well as immune-cell-related proteins. Bulk RNA-seq revealed a dose-dependent attenuation of disease-associated microglial (DAM) and glycolytic gene expression, which is supported by a parallel decrease of glucose uptake and protein levels of Triggering Receptor Expressed on Myeloid cells 2 (Trem2) protein, a major immune receptor involved in DAM activation of microglia. In contrast, DAM activation around residual plaques remains high, regardless of treatment dose. In addition, microglia surrounding residual plaques display a dose-dependent increase in microglial clustering and a selective increase in antigen-presenting and immune signalling proteins. These findings demonstrate that chronic early intervention by an anti-amyloid immunotherapy leads to a dose-dependent decrease in plaque formation, which is associated with lower brain-wide microglial DAM activation and neurodegeneration. Microglia at residual plaques still display a combined DAM and antigen-presenting phenotype that suggests a continued treatment response.

**Supplementary Information:**

The online version contains supplementary material available at 10.1186/s13024-025-00878-1.

## Introduction

Alzheimer’s disease (AD) patients present with the deposition of aggregation-prone amyloid-β-peptide (Aβ). In patients suffering from autosomal dominant AD, it has been shown that aggregation of Aβ is initiated approximately 25 years before symptom onset, triggering a pathological cascade of Tau aggregation, neurodegeneration and neuroinflammation that ultimately leads to cognitive decline [1, 2]. Based on the pioneering work by Schenk and colleagues [3] therapeutic antibodies, such as Aducanumab, Lecanemab and Donanemab were developed that target aggregated forms of Aβ. In phase III clinical trials, these antibodies have been shown to significantly reduce amyloid plaque burden upon chronic treatment in patients and, for the latter two, to modestly slow cognitive decline [4–6]. In addition, pathological Tau is reduced and patients with low Tau burden have been reported to show stronger clinical effects [5] supporting the idea that early amyloid removal can alter the course of the pathological cascade. Anti-Aβ immunotherapy is the first and currently only disease-modifying treatment for patients with early symptoms of AD or mild cognitive impairment (MCI). However, beyond established clinical outcomes, the effects of early and long-term immunotherapy remain largely unknown.

Aβ-targeting antibodies remove Aβ by recruiting the brain’s immune system, namely microglia, inducing a transient immune response that results in the phagocytosis of aggregated Aβ [7, 8]. This treatment appears to bypass many of the risks associated with active immunization [9] but is associated with side effects such as brain oedema and haemorrhages (detected with MRI as amyloid related imaging abnormalities (ARIA)), which had fatal consequences in rare cases [10]. The mechanisms of these side effects are currently poorly understood, but are potentially linked to immune cell activation [11, 12]. To improve the safety of anti-Aβ antibody treatment, it is therefore important to gain a deeper understanding of the long-term effects of anti-Aβ antibody treatment on microglia.

Microglia can exist in transient states that are defined by transcriptional signatures, which signify their functions. Under pathological conditions, such as the accumulation of aggregated Aβ, microglia transition from a homeostatic state to a disease-associated microglia (DAM) state that is characterised by increased metabolic activity, proliferation, chemotaxis and phagocytosis [13, 14]. This transition relies in part on the presence of microglial Triggering Receptor Expressed on Myeloid cells 2 (TREM2). Loss of function variants of TREM2 are associated with an increased risk for late-onset AD (LOAD) [15, 16] and microglia that lack TREM2 do not convert into the full DAM state and are less capable of dealing with Aβ-related challenges [17–22]. Conversely, higher CSF TREM2 is associated with slower rates of Aβ deposition, reduced cortical shrinkage and diminished cognitive decline [23–26]. The microglial TREM2-dependent transition into the DAM state is therefore thought to be a protective response to fight Aβ deposition and is currently explored for the development of TREM2 agonists [13].

Lack of Trem2 was previously shown to impact the efficacy of anti-Aβ antibody treatment *ex vivo* [27] suggesting that microglial function can influence the efficacy of treatment. Vice versa, Aβ-targeting immunotherapies can affect microglia, which has already been considered in early immunotherapy studies to a limited extent [7, 8, 28]. In recent years, studies have shown that short-term treatment (< 4 weeks) with high doses of anti-Aβ antibodies in mouse models of amyloidosis results in a limited reduction of Aβ load, but is associated with increased expression of homeostatic genes and reduced expression of DAM genes [29, 30]. Studies on long term-treatment with anti-Aβ antibodies (> 8 weeks) are quite inconsistent and report limited to extensive Aβ removal depending on dose, mouse model, treatment start and duration [8, 30–40]. More consistently, these studies report increased clustering of microglia around plaques [29, 31, 33, 36, 37, 41–45]. However, to date, only few studies have sought to provide unbiased insights into the long-term treatment effects on the transcriptional and functional state of microglia or potential biomarker readouts for monitoring the treatment response [29, 30, 34, 46].

Here, we investigate the effect of early chronic anti-Aβ antibody (referred to as anti-Aβ) treatment on Aβ deposition and microglial activation in APP-SAA knock-in (KI) mice [47]. After 16 weeks of treatment, starting at an early phase of plaque deposition, anti-Aβ reduced plaque load, with a higher efficiency for diffuse fibrils, and concomitantly reduced neurite dystrophy in a dose-dependent manner. This was accompanied by a reduction in biomarkers of neurite dystrophy and microglial activation in CSF. Bulk RNA sequencing and *in vivo* PET-imaging, together with proteomic analyses, demonstrated a dose-dependent global attenuation of DAM activation and glycolysis, but identified increased expression of genes associated with antigen presentation. When analysing microglia at plaques that formed despite the early intervention treatment (residual plaques), we found increased clustering and DAM activation, and in addition, a specific induction of MHC-II and Galectin-3 in a dose-dependent manner.

## Methods

### Mice

**Fig. 1:**
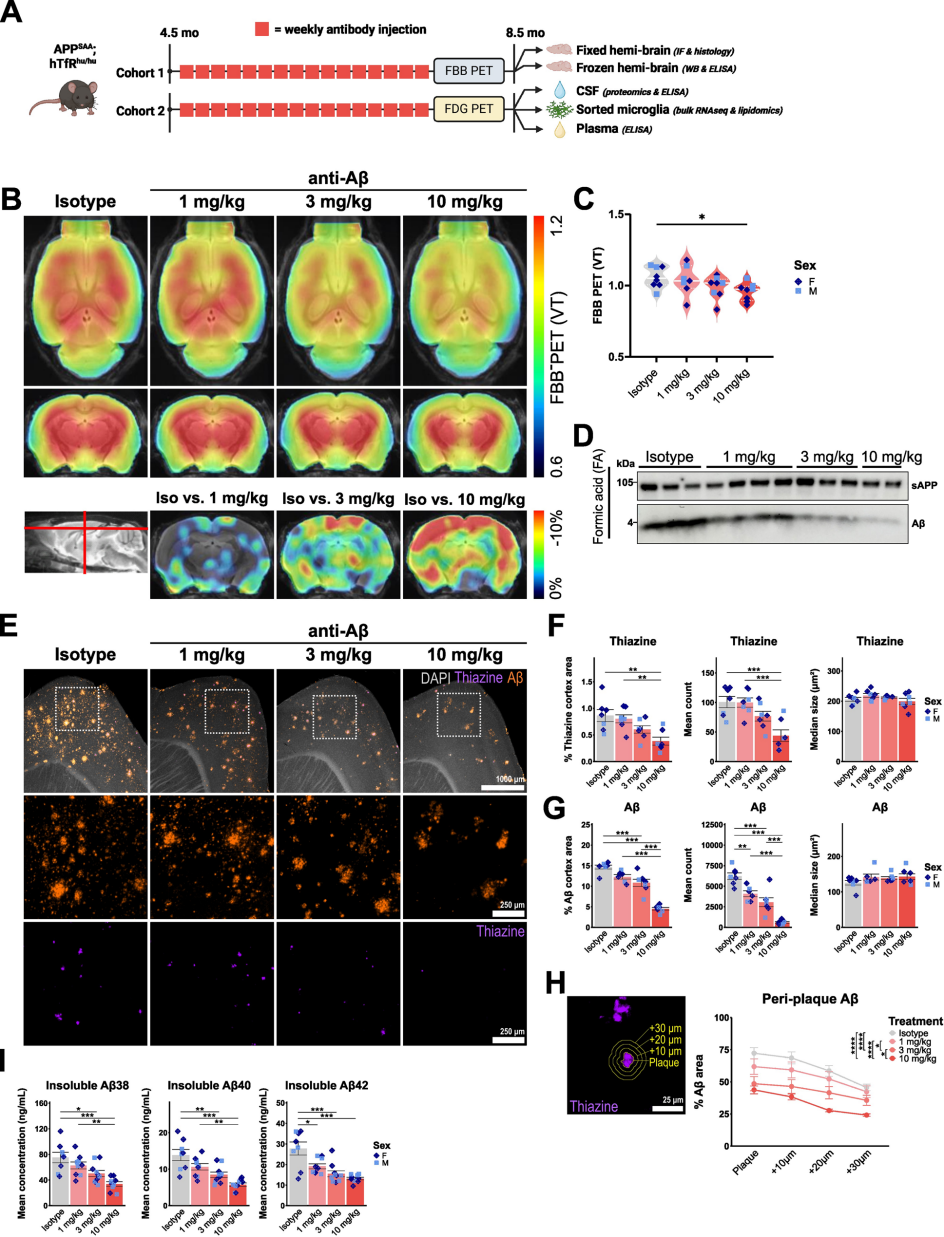
Chronic anti-Aβ treatment reduces Aβ levels in a dose-dependent manner. (**A**) Schematic of the study design and collected materials of anti-Aβ or isotype (4D5) antibody treatment cohorts. (**B**) Axial and coronal FBB-PET distribution volume (VT), and coronal FBB-PET (percent change from isotype) per group projected upon a standard magnetic resonance imaging (MRI) T1 atlas. (**C**) Quantification of FBB-PET (VT). (**D**) Western blot showing soluble (s)APP and Aβ in the formic acid (FA) extracted brain fraction. (**E**) Representative immunofluorescent images of sagittal cortical sections showing DAPI (grey), thiazine (purple) and Aβ (3552 antibody, orange) with insets showing thiazine and Aβ. (**F**) Quantification of percent cortical thiazine^+^ plaque area size and number. (**G**) Quantification of percent cortical pan-Aβ (3552) area size and number. (**H**) Example of concentric plaque regions of interest (ROIs) and quantification of Aβ (3552) signal in these ROIs. (**I**) ELISA quantification of FA extracted insoluble Aβ. *: P < 0.05; **: P < 0.01; ***: P < 0.001, ****: P < 0.0001. One-way ANOVA with Tukey’s post hoc test (**C**, **F**, **G**, **H**, **I**). Schematic (**A**) was created with BioRender.com. For (**B**, **C**): *n* = 5 f, 3 m per group. For (**F**, **G**, **H**): isotype *n* = 5 f, 3 m, 1 mg/kg *n* = 5 f, 4 m, 3 mg/kg *n* = 5 f, 5 m, 10 mg/kg n = 5 f, 4 m. For (**I**), isotype *n* = 5 f, 3 m, 1 mg/kg *n* = 5 f, 4 m, 3 mg/kg *n* = 5 f, 5 m, 10 mg/kg *n* = 5 f, 4 m

All animal experiments were approved by the Ethical Review Board of the Government of Upper Bavaria. Mice were group housed with littermates in standard sized, individually ventilated cages on a 12-hour light/dark cycle, with enriched environment and ad libitum access to food and water. Both sexes were used for all experiments. APP-SAA^ki/ki^ x hTfR^ki/ki^ (APP-SAA x hTfR KI) mice [47–49] were acquired from Denali Therapeutics or bred in our mouse facility and maintained on a C57BL/6J genetic background. hTfR KI was bred into these mice in preparation of future antibody dosing studies that exploit antibody transport vehicle (ATV) technology, but was not investigated in the current study and was previously not found to impact microglia phenotypes in response to Aβ [49] (and Fig. S1). Shipped mice were acclimated for a minimum of two weeks before entering experiments. For anti-Aβ treatment, the chimeric anti-Aβ antibody Aducanumab was used, which contains a mouse IgG2 Fc domain with full effector function [31]. For isotype control, the antibody 4D5 was used, which has a mouse IgG2 Fc domain and is raised against human HER2, a non-existent target in mice [50]. Mice were randomly assigned to a treatment arm and two mouse cohorts underwent treatment. For cohort 1, isotype antibody was dosed at 1 mg/kg and for cohort 2, 10 mg/kg isotype antibody was dosed at 10 mg/kg. Anti-Aβ antibody was dosed at 1 mg/kg, 3 mg/kg or 10 mg/kg. Mice were treated from the average age of 4.48 ± 0.12 months (cohort 1) or 4.68 ± 0.14 months (cohort 2) via weekly intraperitoneal (i.p.) injection of antibody, which was thawed at 4 °C and diluted with phosphate-buffered saline (PBS). Mice from cohort 1 underwent FBB-PET and mice from cohort 2 were subjected to FDG-PET at 8 months of age. Mice were sacrificed by cardiac perfusion 7 days after the last antibody injection, at an average age of 8.26 ± 0.13 months (cohort 1) or 8.62 ± 0.15 months (cohort 2). From cohort 1, one hemibrain was fixed for immunofluorescent staining and another snap frozen for protein extraction (Fig. 1A). From cohort 2, terminal cerebrospinal fluid (CSF), blood plasma and microglia were collected for microglial RNA-seq and lipidomics, and CSF proteomic analysis.

### Small animal PET/MRI

All rodent PET procedures followed an established standardised protocol for radiochemistry, acquisition times, and post-processing [51] which was transferred to a novel PET/MRI system [52]. In brief, [^18^F]-FBB-PET (florbetaben) and [^18^F]-FDG-PET (fluorodeoxyglucose) were used to measure fibrillar amyloidosis and glucose metabolism respectively after antibody treatment. We studied PET images of 8.12 ± 0.13-month-old APP-SAA mice (*n* = 32) for FBB-PET and 8.48 ± 0.20-month-old APP-SAA mice (*n* = 35) for FDG-PET using at least *n* = 8 per treatment group and tracer. All mice were scanned with a 3T Mediso nanoScan PET/MR scanner (Mediso Ltd., Budapest, Hungary) with a triple-mouse imaging chamber. Isoflurane anaesthesia was applied for all PET experiments (1.5% at time of tracer injection and during imaging; delivery 3.0 L/min). Two 2-minute-long anatomical T1 MR scans (sagittal and axial) were performed after tracer injection (head receive coil, matrix size 96 × 96 × 22 mm^3^, voxel size 0.24 × 0.24 × 0.80 mm^3^, repetition time 677 ms, echo time 28.56 ms, flip angle 90°). Injected dose was 13.1 ± 2.1 MBq for [^18^F]-FBB and 19.1 ± 1.5 MBq for [^18^F]-FDG delivered in 200 µl saline via venous injection. PET emission was recorded in a dynamic 0–60 min window for FBB-PET and in a static 30–60 min window for FDG-PET. List-mode data within 400–600 keV energy window were reconstructed using a 3D iterative algorithm (Tera-Tomo 3D, Mediso Ltd., Budapest, Hungary) with the following parameters: matrix size 55 × 62 × 187 mm^3^, voxel size 0.3 × 0.3 × 0.3 mm^3^, 8 iterations, 6 subsets. Decay, random, and attenuation correction were applied. The T1 image was used to create a body-air material map for the attenuation correction. Framing for FBB-PET was 6 × 10 s, 6 × 30 s, 6 × 60 s, 10 × 300 s.

All analyses were performed by using PMOD software (version 3.5, PMOD Technologies, Basel, Switzerland). To normalise FBB-PET data we generated V_T_ images with an image-derived input function [53, 54] using the methodology described by Logan et al. implemented in PMOD [55]. The plasma curve was obtained from a standardised voxel of interest (VOI) placed in the myocardial ventricle. A maximum error of 10% and a V_T_ threshold of 0 were selected for modelling of the full dynamic imaging data. Normalization of the injected activity for FDG-PET was performed by generating standardised uptake values (SUV), reflecting the common read-out in clinical setting. A cortical volume-of-interest (comprising 40.9 mm^3^) was selected and served for extraction of FBB-PET values. FDG-PET values were extracted from a bilateral entorhinal VOI (comprising 13.0 mm^3^) which was delineated by regions of the Mirrione atlas [56].

### Mouse brain, CSF, and plasma sampling

CSF collection was performed as previously described from treatment cohort 2 [57]. Briefly, mice were anesthetised using a mix of medetomidine (0.5 mg/kg), midazolam (5 mg/kg), and fentanyl (0.05 mg/kg) (MMF) injected i.p. After complete anaesthesia, mice were head-fixed in a stereotaxic frame and the cisterna magna was surgically exposed. The dura was punctured using a borosilicate glass capillary (Sutter, B100-75-10) attached to medical-grade tubing and CSF was gently extracted by applying a negative pressure on the tubing using a syringe (equipped with a 28G needle). CSF samples were deposited from the capillary into protein Lo-Bind tubes (Eppendorf, 0030108094) and kept on ice until centrifugation at 2000 g for 10 min at 4 °C to pellet any red blood cells and visually check for contamination. After CSF collection, blood was extracted via cardiac puncture using a syringe, inserted into Microvette^®^ 500 EDTA K3E tubes (Sarstedt, 20.1341.100), slowly inverted 10 times and kept on ice. Within 1 h, blood was centrifuged at 12700 rpm at 4 °C for 10 min in a tabletop centrifuge. Plasma was then transferred to a protein Lo-Bind tube and snap-frozen. Mice were perfused via cardiac puncture with ice-cold PBS. For cohort 1, brains were split into two hemispheres and one hemisphere was fixed in 4% paraformaldehyde (PFA) with 0.05% NaN_3_ for 48 h. The other hemisphere was snap-frozen in liquid nitrogen and stored at -80 °C. For cohort 2, brains were kept in Hanks’ buffered salt solution with Ca^2+^ and Mg^2+^ (HBSS) (Gibco, 14025092) + 7 mM HEPES (Gibco, 15630080) + 2x GlutaMAX (Gibco, 35050061) on ice until proceeding with microglia isolation.

### Immunofluorescence staining of mouse brain

50-µm brain sections were cut using a vibratome and stored in 15% glycerol + 15% ethylene glycol in PBS for 2 days at 4 °C, before transferring them to a -20 °C freezer for long-term storage. For immunostaining, free-floating sections were washed 5x in PBS on a shaker to remove storage medium. Antigen retrieval was performed in citrate buffer (pH 6) or Tris-EDTA buffer (pH 8 or pH 9) at 80–95 °C for 30 min, depending on the antibody. After antigen retrieval, sections were cooled down to room temperature (RT), briefly washed in PBS and incubated in 10% normal donkey serum (NDS) in PBS + 0.3% Triton X-100 (blocking solution) on a shaker for 1–1.5 h. Section were incubated overnight in blocking solution containing primary antibodies. The next day, sections were washed 3x in PBS + 0.3% Triton X-100 and incubated in secondary antibodies in blocking solution for 1–2 h. In case of co-staining with Thiazine Red (Morphisto, 12990.001), the dye was added to the secondary solution, sections were washed 3x in PBS + 0.3% Triton X-100. For Methoxy-X04 (MX-04, Tocris, 4920) co-staining, sections were incubated in 50% EtOH in PBS with MX-04 for 30 min at RT, washed 5 min in 50% EtOH in PBS, and washed 3x in PBS. In case of HS169 (courtesy of Peter Nilsson, Linsköping University, Sweden) staining, dye was incubated 1:2500 in PBS for 15 min and washed 3x in PBS. If applicable, 40,6-diamidino-2-phenylindole (DAPI) was added to the secondary antibody solution (1:1000). Sections were mounted onto Superfrost Plus slides with ProLong Gold antifade reagent (Thermo Fisher, P36980) or Fluoromount-G (Thermo Fisher, 00-4958-02). After 24 h of drying, slides were stored at 4 °C.

**Table:** 

Primary antibody	Concentration	Catalogue number	Company
Rabbit anti-Aβ (3552)	3.7 µg/mL (1:1000)	n/a	See ref [58]
Mouse anti-Aβ (NAB288)	n/a, (1:500)	2450	Cell Signaling Technology
Rabbit anti-IBA1	n/a, (1:500)	019-19741	Wako
Guinea pig-anti-IBA1	2 µg/mL (1:500)	234 308	Synaptic Systems
Goat anti-APOE (HJ6.3/b)	n/a, (1:300)	n/a	See ref [59]
Rat anti-LAMP1 (1D4B)	1 µg/mL (1:500)	121,602	Biolegend
Rabbit anti-GFAP (Dako)	n/a, (1:500)	GA52461-2	Agilent
Sheep anti-TREM2	1.3 µg/mL (1:150)	AF1729	R&D Systems
Rat anti-CD68 (FA-11)	1 µg/mL (1:500)	1,370,002	Biolegend
Rat anti-MHC Class II (I-A/I-E)	1 µg/mL (1:500)	14-5321-82	Thermo Fisher Scientific
Goat anti-Galectin 3	0.4 µg/mL (1:500)	AF1197	Cell Signaling Technology
Rabbit anti-PU.1 (9G7)	n/a (1:500)	2258	Cell Signaling Technology
Rabbit anti-Laminin	25 µg/mL (1:200)	L9393	Sigma
Rabbit anti-P2RY12	1 µg/mL (1:200)	AS-55,043 A	Anaspec

**Table:** 

Secondary antibody	Concentration	Catalogue number	Company
Donkey anti-rabbit Alexa Fluor Plus 488 IgG (H + L)	(1:1000)	A32790	Invitrogen
Donkey anti-rabbit Alexa Fluor Plus 647 IgG (H + L)	(1:1000)	A32790	Invitrogen
Donkey anti-mouse Alexa Fluor Plus 488 IgG (H + L)	(1:1000)	A32766	Invitrogen
Donkey anti-mouse Alexa Fluor Plus 647 IgG (H + L)	(1:1000)	A32787	Invitrogen
Donkey anti-rat Alexa Fluor Plus 647 IgG (H + L)	(1:1000)	A32795	Invitrogen
Donkey anti-goat Alexa Fluor Plus 647 IgG (H + L)	(1:1000)	A32849	Invitrogen
Donkey anti-sheep Alexa Fluor Plus 647 IgG (H + L)	(1:1000)	A21448	Invitrogen
Donkey anti-guinea pig Alexa Fluor Plus 647 IgG (H + L)	(1:1000)	A21450	Invitrogen

### Prussian blue staining of haemosiderin deposits in mouse brain

For quantification of haemosiderin deposits, slides were mounted onto Superfrost Plus slides and dried for 2 h at room temperature (RT). Slides were rehydrated in PBS and incubated in Prussian blue solution (2 g potassium hexacyanoferrate (II) trihydrate (Sigma, P9387) in 100 mL dH_2_O) for 20 min and in 0.1% Nuclear Fast Red solution (Morphisto, 10264.00500) for 5 min and washed in dH_2_O. Slides were dehydrated from 70 to 100% EtOH and mounted using VectaMount Express Mounting Medium (Vector Labs, VEC-H-5700). The number of Prussian Blue deposits was quantified from 5 brain sections of each mouse by stereology using the Leica DMi8 fluorescence microscope. Images of deposits were acquired using a 40x air lens (0.65 NA, Leica). Area and number of observed deposits was quantified from images using Fiji [60].

### Microscopy and image acquisition

Epifluorescence images were acquired with a Leica DMi8 equipped with a mercury lamp (EL6000, Leica) using a 20x air lens (0.4 NA, Leica) or an Olympus VS200 Slideview slide scanner using a 20x air lens (0.8 NA, 0.274 μm/pixel). Leica scanned tiles were acquired using the Leica Application Suite X software using an overlap of 10% per image and a resolution of 1024 × 1024 (0.651 × 0.651 μm per pixel). Confocal images were acquired with a 63x oil immersion lens (1.4 NA, Zeiss), using a Zeiss LSM800 confocal microscope and the ZEN 2.5 Zeiss software package, at a resolution of 2048 × 2048 (0.0495 × 0.0495 μm per pixel).

### Quantification of plaque number and microglia/plaque association

Image analysis was conducted blinded using a semi-automated ImageJ pipeline, where the user draws the outline of the region of the brain in each image to be analysed and inputs Gaussian filter values and thresholds for each channel. For each image, the pipeline then automatically applies a difference-of-Gaussian filter using Clij2 [61], followed by automated thresholding and subsequently measures total area and intensity of the selected channels. For individual plaque analysis, the total plaque region of interest (ROI) is split into individual ROIs, then using the ROI Manager, each ROI is given a unique name and subsequently area and intensity are measured for each plaque. For concentric ring analysis, the plaque ROI is enlarged and using logical operations (XOR) the original ROI is subtracted from the enlarged ROI to generate concentric rings with a user defined increase in size around the original selection (here 3 × 10 μm). Each concentric ring is given the same name as the original ROI they were generated from + a suffix to denote its increase in size. For each of these rings and the plaque ROIs, the total ROI size as well as selected channel area and intensity within these ROIs is measured. To quantify the area that a selected channel occupies in the vicinity of each plaque specifically within microglia, a threshold for Iba1 is set to obtain an ROI for the entirety of microglia. Then, using logical operators with the ROI manager (AND), ROIs corresponding to microglia colocalizing with plaque ROIs and concentric rings are obtained. Lastly, the total ROI size as well as selected channel area and intensity within these ROIs is measured. For each processed image a.csv file is created, which was subsequently processed, analysed and plotted using R (4.1.1) and R Studio (2024.09.0 + 375) [62]. For percent area calculations, thresholded signal area was divided by the total ROI area and multiplied by 100.

### 3D evaluation of plaque morphology and microglial clustering around plaques

For the evaluation of plaque size, sphericity and proximity of microglia and Aβ to plaques, 5 plaques per mouse were picked randomly and 63x confocal z-stacks were acquired along the cortex (z-distance 1.7 μm). First, images were deconvolved using point spread functions generated with the PSF generator Fiji plugin *(*Hagai Kirshner and Daniel Sage, Biomedical Imaging Group at EPFL) with the Born & Wolf optical model and 10 iterations of Richardson-Lucy deconvolution with the CLIJx plugin [61]. Then, using an automated Fiji script, a 3D difference-of-Gaussian filter was applied and images were made isotropic using Clij2. Then, using the 3D ROI manager [63] individual ROIs were imported from each microglia nucleus (based on PU.1^+^ nuclei) or Aβ (3552), from each thiazine^+^ plaque (excluding objects touching the image edges, as well as top and bottom z-slices) and from the total image volume. 3D measurements of each plaque (volume, sphericity, etc.), distance of each PU.1^+^ nucleus or Aβ ROI to each plaque and colocalization between each plaque and the total volume of microglia were obtained using 3D manager built-in functions. These measurements were exported as.csv files and further processed, analysed and plotted using R (4.1.1) and R Studio (2024.09.0 + 375). Measurements of 5 plaques per mouse were averaged and images where PU.1^+^ ROI separation was not achieved were excluded. Representative 3D isotropic images were made using napari [64].

### Protein extraction

Whole hemispheres were lysed following a previously published protocol [65] and kept at 4 °C during all steps. Briefly, hemispheres were lysed in DEA buffer (0.2% diethylamine in 50 mM NaCl, pH 10, and protease inhibitor mix (Sigma, P8340) using the Precellys homogeniser in 2-mL Tissue Homogenizing CKmix tubes (Precellys, P000918-LYSK0-A). Lysate was centrifugated 10 min at 4000 g and supernatants were ultracentrifugated at 100 000 g before collection. Samples were neutralised by adding 10% of 0.5 M Tris-HCl buffer (pH 6.8) to each sample (DEA fraction). Remaining pellets in Precellys tubes were lysed in RIPA buffer (20 mM Tris-HCl (pH 7.5), 150 mM NaCl, 1 mM EDTA, 1% NP-40, 1% sodium deoxycholate, 0.1% SDS, and protease inhibitor mix). RIPA lysates were centrifuged 10 min at 4000 g, and the supernatants were ultracentrifuged at 100 000 g for 60 min before collection (RIPA fraction). The remaining material in Precellys tubes was resuspended in 70% formic acid with protease inhibitor mix and sonicated for 7 min. Samples were centrifuged at 20 000 g for 20 min and collected supernatant was diluted 1:20 in pH-neutralizing 1 M Tris-HCl buffer (pH 9.5) (FA fraction). Protein concentration was measured using Pierce Bicinchoninic acid (BCA) assay (Thermo Scientific, 23225).

### Enzyme-linked immunosorbent assay (ELISA)

Aβ levels in FA fraction and CSF were determined using the Meso Scale Discovery (MSD) platform and the V-PLEX Plus Aβ Peptide Panel 1 (6E10) Kit (Meso Scale Discovery, K15200G). FA samples were diluted 1:10 in dilution buffer (Diluent Assembly 9), CSF was diluted 1:60. Cxcl10/IP-10 levels in DEA fraction were measured using the MSD U-PLEX Mouse IP-10 Assay (Meso Scale Discovery, K152UFK) at a dilution of 1:2.

TREM2 levels in DEA and RIPA fractions, as well as CSF, were measured using the MSD platform as described previously [66]. Briefly, MSD-gold Streptavidin-coated 96-well plates (Meso Scale Discovery, L15SA-1) were coated in 3% bovine serum albumin (BSA) + 0.05% Tween 20 in PBS (blocking buffer) overnight at 4 °C. Sample is diluted in 1% BSA + 0.05% Tween 20 in PBS + protease inhibitor mix (Sigma, P8340), 1:10 for DEA, 1:2 for RIPA, and 1:35 for CSF. The plate is incubated with 25 µL/well capture antibody in blocking buffer for 90 min, followed by 120 µL/well sample for 2 h, detection antibody 50 µL/well for 60 min and SulfoTAG antibody 25 µL/well for 60 min at 600 rpm at RT. In between each incubation the plate is washed 3x with 0.05% Tween 20 in PBS. Before read-out, the plate is washed 2x in PBS, 150 µL/well MSD Read buffer T (Meso Scale Discovery, R92TC-1) is added and is read immediately.

**Table:** 

Capture Antibody	Concentration	Catalogue number	Company
Goat anti-Trem2 biotinylated	0.125 µg/mL (1:800)	BAF1729	R&D Systems
**Detection Antibody**			
Rat anti-Trem2 (5F4)	1 µg/mL (1:1000)	n/a	In-house, see ref [27]
Rabbit anti-Trem2 (HL1738)	n/a (1:10.000)	MA5-31267	Thermo Fisher
**SULFO-TAG Antibody**			
Goat anti-rat-SULFO-TAG	0.5 µg/mL (1:1000)	R32AH-1	Meso Scale Discovery
Goat anti-rabbit-SULFO-TAG	0.5 µg/mL (1:1000)	R32AB	Meso Scale Discovery

### Western blot

4x Laemmli Buffer (Biorad, 1610747) + 10% β-mercaptoethanol was added to all samples. For Aβ immunoblotting DEA and FA lysates were loaded on Novex WedgeWell 10 to 20%, Tris-Tricine, 1.0 mm gels (Thermo Fisher, EC66255) and run in 1x Tris-Tricine-SDS buffer. For Trem2 and APP analysis, samples were run on 12% freshly cast Tris-Glycine gels in Tris-Glycine-SDS buffer. Protein was transferred to nitrocellulose membrane using wet transfer in Tris-Glycine buffer (25 mM Tris, 192 mM glycine, pH 7.5). Membranes were boiled in PBS for 15 min before blocking 1–2 h in 0.2% I-Block Protein-Based Blocking Reagent (Applied Biosystems, T2015) and 0.1% Tween 20 in Tris-buffered saline (TBS) (blocking buffer). Membranes were incubated in primary antibody in blocking buffer O/N at 4 °C while shaking. After 3 × 10 min washes in TBS + 0.05% Tween 20 (TBS-T) membranes were incubated with secondary antibody in blocking solution for 1 h at RT while shaking. Membranes were developed using Pierce ECL Western Blotting-Substrate (Thermo Scientific, 32106) and signals visualised using autoradiographic development using Fujifilm Medical X-ray Film Super RX-N (Fujifilm, 47410) or using the Amersham ImageQuant 800.

**Table:** 

Primary antibody	Concentration	Catalog number	Company
Rat anti-Aβ (2D8)	1:50 from hybridoma supernatant	n/a	n/a
Rat anti-TREM2 (5F4)	1 µg/mL (1:1000)	n/a	In-house, see [27]
Rabbit-anti-APP (Y188)	0.384 µg/mL (1:1000)	ab32136	Abcam

**Table:** 

Secondary antibody	Concentration	Catalog number	Company
Goat anti-Rat IgG (H/L): HRP	(1:1000)	5204 − 2504	Biorad
Goat anti-Rabbit IgG (H/L): HRP	(1:1000)	5196 − 2504	Biorad

### Magnetic-activated microglia sorting (MACS) from mouse brain

Prior to microglia isolation, meninges were removed by gently rolling brains on a clean piece of Whatman paper. Cerebellum, pons and olfactory bulb were removed, the two hemispheres were split and any remaining meninges were removed with Dumont forceps using a dissection microscope. Each hemisphere was cut into pieces using a scalpel and brain tissue was dissociated following manufacturer’s instructions using the Neural Tissue Dissociation Kit (P) (Miltenyi, 130-092-628) supplemented with 5 µM Actinomycin D (Cell Signaling Technology, 15021) and 2 µM Anisomycin (Cell Signaling Technology, 2222) in gentleMACS C-tubes (Miltenyi, 130-096-334) using a gentleMACS Dissociator (Miltenyi). Homogenised tissue was run through a 40-µm cell strainer (Corning, 352340) and pelleted by centrifugation. Pellets were resuspended in HBSS with 0.25% fatty acid-free BSA (Sigma-Aldrich, A8806), incubated with magnetic Cd11b^+^ MicroBeads (Miltenyi, 130-093-634) and run twice over MS columns (Miltenyi, 130-042-201). Viable cells were counted using trypan blue, aliquoted into tubes, centrifuged, and snap frozen in liquid nitrogen until further processing.

### Sample preparation for mass spectrometry

CSF samples were prepared as described previously [57]. Briefly, a volume of 5 µL CSF was used for proteolytic digestion. Proteins were reduced by the addition of 2 µL of 10 mM dithiothreitol (Biozol, Germany) in 50 mM ammonium bicarbonate and incubated for 30 min at 37 °C. Cysteine residues were alkylated by the addition of 2 µL 55 mM iodoacetamide (Sigma Aldrich, US) and incubated for 30 min at room temperature in the dark. Afterwards, the reaction was quenched by adding another 2 µL of 10 mM dithiothreitol. Proteolytic digestion was performed using a modified protocol for single-pot solid-phase enhanced sample preparation (SP3) [67]. After binding proteins to 40 µg of a 1:1 mixture of hydrophilic and hydrophobic magnetic Sera-Mag SpeedBeads (GE Healthcare, US) with a final concentration of 70% acetonitrile for 30 min at room temperature, the beads were washed four times with 200 µL 80% ethanol. For proteolytic digestion, 0.1 µg LysC and 0.1 µg trypsin (Promega, Germany) were added in 20 µL 50 mM ammonium bicarbonate followed by an incubation for 16 h at room temperature. The magnetic beads were retained in a magnetic rack and the supernatants were filtered with 0.22 μm spin filters (Spin-X, Costar) to remove remaining beads and dried by vacuum centrifugation.

### Liquid chromatography tandem mass spectrometry (LC-MS/MS) of CSF

Dried peptides were dissolved in 20 µL 0.1% formic and 5.5 µL were separated on a nanoElute nanoHPLC system (Bruker, Germany) on an in-house packed C18 analytical column (15 cm × 75 μm ID, ReproSil-Pur 120 C18-AQ, 1.9 μm, Dr. Maisch GmbH) using a binary gradient of water and acetonitrile (B) containing 0.1% formic acid at flow rate of 300 nL/min (0 min, 2% B; 2 min, 5% B; 62 min, 24% B; 72 min, 35% B; 75 min, 60% B) and a column temperature of 50 °C. The nanoHPLC was online coupled to a timsTOF Pro mass spectrometer (Bruker, Germany) with a CaptiveSpray ion source (Bruker, Germany). A Data-Independent Acquisition Parallel Accumulation-Serial Fragmentation (diaPASEF) method was used for spectrum acquisition. Ion accumulation and separation using Trapped Ion Mobility Spectrometry (TIMS) was set to a ramp time of 100 ms. One scan cycle included one TIMS full MS scan with 26 windows with a width of 27 m/z covering a m/z range of 350–1001 m/z. Two windows were recorded per PASEF scan. This resulted in a cycle time of 1.4 s.

### Mass spectrometry data analysis

The software DIA-NN version 1.8.1 was used to analyse the data [68]. The raw data was searched against a one-protein-per-gene database from Mus musculus (UniProt, 21709 entries, download: 2024-02-19) combined with a database of common human contaminations (123 entries) using a library-free search. Trypsin was defined as protease and two missed cleavages were allowed. Oxidation of methionines and acetylation of protein N-termini were defined as variable modifications, whereas carbamidomethylation of cysteines was defined as fixed modification. The precursor and fragment ion m/z ranges were limited from 350 to 1001 and 200 to 1700, respectively. An FDR threshold of 1% was applied for peptide and protein identifications. The mass accuracy and ion mobility windows were automatically adjusted by the software. The match between runs option was enabled.

The statistical analysis was performed with the software Perseus version 1.6.2.3 [69]. First, a one-way ANOVA was used to determine statistically significant differences between the means of the groups. Afterwards, individual Student’s *t*-tests were applied to evaluate proteins with a significant abundance difference between 1, 3, and 10 mg/kg anti-Aβ compared to isotype control treatment. Additionally, isotype control samples were compared with sample from 3-month-old untreated mice. A permutation-based false discovery rate estimation was used with a FDR of 5% at s0 = 0.1 as threshold [70].

### RNA isolation, RT‑qPCR, and library preparation

To prepare for RNA-seq, approximately 100 000 CD11b^+^ microglia isolated by MACS were used for RNA extraction by the RNeasy Plus Micro Kit (Qiagen, #74034). The extracted RNA was then resuspended in nuclease-free water for RNA-seq library preparation. Libraries for 30 total RNA samples were prepared using the Lexogen QuantSeq 3′ mRNA-Seq V2 Library Prep Kit FWD with Unique Dual Indices (Lexogen 193.384) and the UMI Second Strand Synthesis Module, following the manufacturer’s protocol to identify and remove PCR duplicates. In brief, total RNA was used as input for oligo(dT) priming during reverse transcription, followed by RNA removal. Unique Molecular Identifiers (UMIs) were incorporated during second-strand synthesis. The cDNA was purified using magnetic beads, amplified with 18 cycles of PCR, and subsequently purified again. Library quantity and quality were assessed using a TapeStation D1000 ScreenTape (Agilent 5067–5582). Equimolar pooling of libraries was performed, and sequencing reads were generated on one lane of an Illumina NovaSeq X 10B cartridge (75 bp single-end) by SeqMatic (Fremont, CA, USA).

### RNA-seq data analysis

RNA-seq data was processed using nf-core/rnaseq v3.11.2 (10.5281/zenodo.1400710) of the nf-core collection of workflows [71]. Reads were aligned to the GRCm39 release of the mouse genome, and gene annotations were obtained from Gencode M31. To account for the use of UMIs in the library preparation protocol, the following arguments were passed to the STAR aligner (version 2.7.9a [72]),: --alignIntronMax 1,000,000 --alignIntronMin 20 --alignMatesGapMax 1,000,000 --alignSJoverhangMin 8 --outFilterMismatchNmax 999 --outFilterType BySJout --outFilterMismatchNoverLmax 0.1 --clip3pAdapterSeq AAAAAAAA. After alignment, UMIs were extracted with the following regular expression: ^(?P.{6})(?P.{4}).*. As each transcript is only represented by a single sequence, the --noLengthCorrection parameter was passed to the salmon (version 1.10.1 [73]) gene-level quantitation step. The pipeline was executed with Nextflow v23.10.0 [74]. Downstream analysis was performed using R (version 4.4.0) using the limma/voom workflow [75] to fit linear models for each quantifiable gene. Library sizes were estimated using the TMM method [76] and we fit a linear model with treatment group and sex as fixed covariates, and takedown-batch as a random effect with the voomLmFit function from the edgeR R package (version 4.2.0 [75]),. Sample weights were included by setting the sample.weights argument to TRUE. Differentially expressed genes were identified with the eBayes function from the limma R package (version 3.60.0 [77]),, setting the robust = TRUE argument. *P*-values were corrected for multiple-testing according to [78]. Gene set enrichment analyses were performed with the fgsea R package (version 1.30.0 [79]),, with gene sets obtained via the msigdbr package (7.5.1, doi: 10.32614/CRAN.package.msigdbr).

### Lipid extraction

Cell pellets (100 000 MACS-sorted cells) were suspended in 400 µL of a 3:1 butanol/methanol extraction buffer with stable isotope-labeled internal standards and mixed for 5 min at 600 rpm on a plate shaker at room temperature. Plates were stored for one hour at -20 °C and centrifuged at 21 000 g for 5 min at 4 °C. After centrifugation, 200 µL of the supernatant was collected and dried under a continuous stream of nitrogen gas. The dried extracts were reconstituted in 200 µL of LC-MS-grade methanol for subsequent analysis.

### LC-MS analysis of lipids

Lipid analysis was performed using an Agilent Infinity II 1290 UHPLC coupled with a QTRAP 6500 + mass spectrometer. Lipids were analysed in both positive and negative ionization modes and resolved on a UPLC BEH C18 column (150 × 2.1 mm, 1.7 μm, Waters Corp.) at 55 °C with a 0.25 mL/min flow rate, following the buffer and gradient schedule as described previously [80]. Data acquisition, peak integration, and quantification were conducted using MultiQuant (version 3.3, ABSciex) with a minimum signal-to-noise ratio of 5 and at least 8 points across the baseline.

### Statistical analysis

Unless indicated otherwise in the methods, statistical analysis was performed in R studio (R version 4.2.3) [62]. Data are shown with the mean and standard error of the mean (± SEM), unless indicated otherwise. For normally distributed data a one-way ANOVA was applied. Statistical evaluations are displayed as follows: *P < 0.05; **P < 0.01; ***P < 0.001; ****P < 0.0001. Graphs were plotted using the *tidyverse* package and statistical significance was plotted using the *ggsignif* package [81, 82].

## Results

### Chronic anti-Aβ treatment reduces amyloid with greater preference for loosely aggregated fibrils in a dose-dependent manner

APP-SAA KI mice have previously been reported to develop MX-04^+^ plaques with an increased brain Aβ_42/40_ ratio compared to wild-type mice at 4 months of age [47]. To investigate the progression of pathology in APP-SAA KI mice and determine the optimal treatment window for early intervention, we investigated amyloid plaque deposition at 3, 6, and 12 months of age. Immunostaining of brain sections with thiazine red, a dye that binds to core plaques [83] and an Aβ-antibody (3552), which binds to total Aβ [58] indicates that APP-SAA KI mice already show the sporadic appearance of diffuse Aβ fibrils in plaque-like structures as early as 3 months (Fig. S1A, B). Furthermore, these diffuse plaques are already surrounded by Lamp1^+^ neurite dystrophy. Thiazine^+^ dense-core plaques appear at 6 and 12 months together with Trem2^+^ microglia (Fig. S1C, D). Trem2 protein levels increase with age and amyloid plaque accumulation, suggesting a strong induction of DAM activation concomitant with the accumulation of plaques, as observed in other mouse models [84, 85] (Fig. S1E). Interestingly, microglia also appear to cluster around diffuse plaques already starting at 3 months (Fig. S1F). Due to the Austrian mutation [86] plaques of APP-SAA KI mice contain a large amount of Aβ_38_, which is not typically incorporated into amyloid plaques in sporadic AD patients [87, 88]. Most Aβ accumulates in the insoluble (FA) fraction, with limited detection in the soluble (DEA) fraction (Fig. S1G, H). Importantly, the anti-Aβ antibody used for dosing studies in this model (Aducanumab) binds to residues 3–7 of Aβ, which do not overlap with the three dominant mutations inserted into the humanised APP gene (Fig. S1I) [89]. 

To understand the dose-dependent effects of a chronic intervention treatment paradigm and potential dose-dependent effects, APP-SAA KI mice were treated with 1, 3, or 10 mg/kg of anti-Aβ or an isotype antibody (1 or 10 mg/kg) weekly via i.p. injection for 16 weeks (Fig. 1A). To simulate an early intervention paradigm where plaques just start to form, a time point of 4.5 months was chosen to start treatment. The [^18^F]-florbetaben (FBB)-PET [90] signal detected in 8-month-old APP-SAA KI mice is reduced in a dose-dependent manner by anti-Aβ treatment (Fig. 1B, C). The reduction of Aβ is confirmed by Western Blot analysis of the FA brain extract (Fig. 1D). In contrast, sAPP (Fig. 1D), full-length APP and its C-terminal fragments (CTF-α and CTF-β) are not altered in total brain extracts by the antibody treatment, confirming no changes in APP processing upon treatment (Fig. S2A). Immunostaining of saggital brain sections with thiazine and Aβ-antibody (3552), indicates that plaques and fibrils are mainly located in the cortex (Fig. 1E), hippocampus and cortico-amygdala area (COA) (Fig. S2B). Treatment with anti-Aβ reduces both the area and number of thiazine^+^ dense-core plaques, as well as percent Aβ area in a dose-dependent manner in all three brain regions (Fig. 1F, G, and S2C). Interestingly, percent Aβ area and number is reduced more strongly upon anti-Aβ treatment, as shown by immunostaining with a pan-Aβ antibody, as opposed to a reduction in thiazine^+^ dense-core plaque area and number, only seen upon treatment with 10 mg/kg anti-Aβ. This suggests that diffuse Aβ fibrils are more readily prevented from aggregating into plaques. Analysis of percent area covered by Aβ at and around dense-core plaques in concentric rings indicates a dose-dependent reduction in peri-plaque Aβ (Fig. 1H). To investigate whether this could be associated with changes in plaque compactness, we performed confocal imaging of whole plaques and analysis of 3D-reconstructed images. However, no changes are observed in plaque compactness when quantifying the Aβ/thiazine volume ratio or mean distance of Aβ objects to thiazine (Fig. S2D, E). Lastly, whole-brain levels of different Aβ species were quantified using an Aβ-triplex ELISA, which shows a significant dose-dependent reduction at 3 and 10 mg/kg doses in Aβ_38_, Aβ_40_, and Aβ_42_ levels (Fig. 1I). In parallel in CSF, although individual Aβ_38_, Aβ_40_, and Aβ_42_ levels show high between-mouse variability, an increase in the CSF Aβ_42/40_ ratio is observed (Fig. S2F). Overall, these results suggest a significant removal of aggregated Aβ, and prevention of plaque formation in APP-SAA mice upon chronic anti-Aβ treatment, with a higher efficiency for preventing deposition of diffusely aggregated forms of Aβ.

### Chronic anti-Aβ treatment does not worsen microhaemorrhages in APP-SAA KI mice

Clinical use of anti-Aβ antibodies is associated with an increased risk of microbleeds, the cause of which has been hypothesised to be linked to the treatment-induced immune response, as well as amyloid deposition in cerebral blood vessels [11, 12]. To investigate vascular amyloid pathology in the APP-SAA KI mouse, we used HS169, a compound that binds to both diffuse and fibrillar plaques and vascular Aβ deposits [91]. We find that APP-SAA KI mice show cerebral amyloid angiopathy (CAA)-like Aβ aggregation in meningeal and cerebral blood vessels at the age of 8.5 months (Fig. S3A). By quantifying HS169 area, we confirm a dose-dependent reduction in percent cortical Aβ area, but observe no significant changes in HS169 co-localizing with vascular marker laminin (α1) upon treatment (Fig. S3B, C). Previous reports also described that anti-Aβ antibody treatment in mouse models can lead to an increase in microbleeds [30, 33]. To investigate whether chronic anti-Aβ treatment in APP-SAA KI mice is associated with microbleeds in a dose-dependent manner, Prussian blue staining of haemosiderin was analysed. Surprisingly, evidence of past microbleeds, in the forms of Prussian blue-positive microglial cell-shaped foci, is observed in sections of all treatment conditions, including treatment with the isotype control (Fig. S3D). However, no significant changes are observed in foci count or percent area across sections with anti-Aβ treatment (Fig. S3E). To confirm whether this effect could be associated with antibody treatment in general, control sections of untreated 3-, 6-, and 12-month-old APP-SAA KI mice were analysed (Fig. S3F). Interestingly, these mice also show Prussian blue foci without antibody treatment at 6 and 12 months, but not at 3 months (Fig. S3F). These results suggest that the APP-SAA KI mouse model develops spontaneous microbleeds, which may be related to the presence of CAA, which appear not worsened by antibody treatment as measured by Prussian blue staining.

### CSF proteome changes indicate reduced immune activation and neuropathology after chronic anti-Aβ treatment

**Fig. 2:**
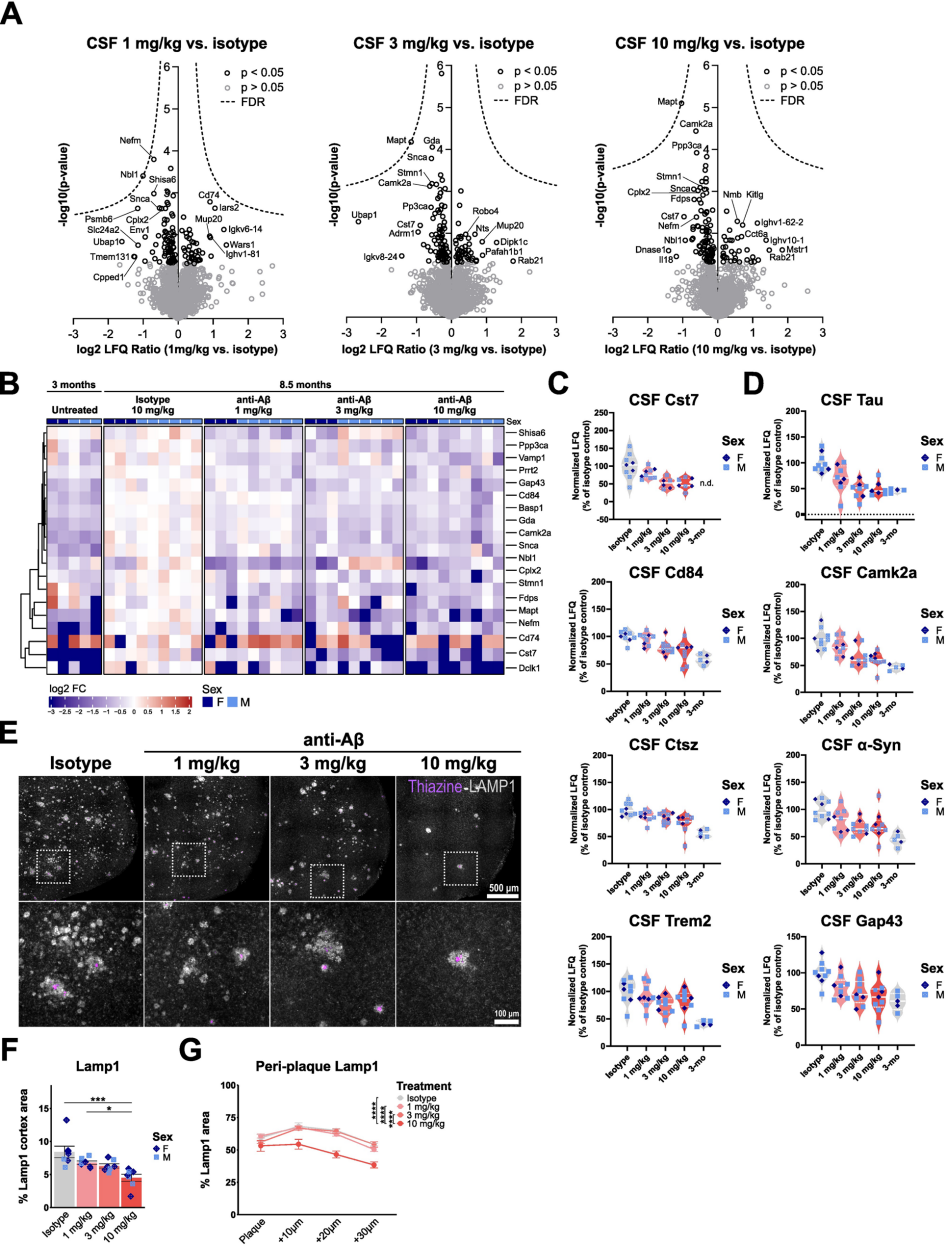
CSF proteome changes indicate reduced neuropathology and immune activation after chronic anti-Aβ treatment. (**A**) Volcano plots showing upregulated and downregulated proteins in CSF. Each volcano shows changes in comparison to isotype-treated mice. (**B**) Heatmap showing unbiased clustering of top 20 changed proteins relative to isotype control-treated mice (cut-off *p* < 0.05, log2FC < -0.5 or > 0.5). (**C**) Normalised LFQ plots of microglial and (**D**) neuronal proteins relative to isotype. (**E**) Representative fluorescent images showing thiazine (purple) and Lamp1 (grey) in sagittal cortical sections. (**F**) Quantification of Lamp1% area in the cortex. (**G**) Quantification of Lamp1 in concentric rings in and around thiazine plaques. **: *P* < 0.01; ***: *P* < 0.001; ****: *P* < 0.0001. One-way ANOVA with Tukey’s post hoc test (**F**, **G**). For (**A**, **B**, **C**): untreated *n* = 2 f, 3 m, isotype *n* = 3 f, 6 m, 1 mg/kg *n* = 3 f, 6 m, 3 mg/kg *n* = 3 f, 6 m, 10 mg/kg *n* = 3 f, 6 m. For (**F**, **G**): isotype *n* = 5 f, 3 m, 1 mg/kg *n* = 5 f, 4 m, 3 mg/kg *n* = 5 f, 5 m, 10 mg/kg *n* = 5 f, 4 m

CSF biomarkers are routinely used in clinical practice to diagnose AD [92]. In addition, antibody treatments in clinical trials helped to identify novel biomarkers that can predict better or worse disease outcomes and could potentially be used to monitor target engagement upon anti-Aβ antibody treatment in patients [93]. To investigate potential CSF biomarkers of anti-Aβ treatment, we analysed terminally-collected CSF of treated mice using liquid chromatography tandem mass-spectrometry (LC-MS/MS) and shotgun proteomics with label-free quantification. CSF from 3-month-old untreated APP-SAA mice was included as a baseline/pre-treatment comparison. When comparing 8.5-month-old isotype treated mice to 3-month-old untreated APP-SAA KI mice, several inflammatory marker proteins such as Trem2, Lag3, Lyz2, and Sod2 as well as the neurodegenerative marker Tau (Mapt) show an increased abundance, which is partially attenuated with 10 mg/kg anti-Aβ treatment (Fig. S4A, B). Comparing individual treatment doses to isotype control, several disease-related proteins show decreased abundances, which are however not significant after FDR correction (Fig. 2A, B). The immune cell-related proteins Cst7, Cd84, Ctsz and Trem2 show a dose-dependent response (Fig. 2C). ELISA of CSF show a sex-specific effect with sTrem2 levels decreasing significantly only in males (Fig. S4C, D). In terminally-collected plasma no significant treatment-dependent differences in sTrem2 concentrations are observed (Fig. S4E). Interestingly, LC-MS/MS analysis also identifies neuronal proteins in CSF, such as Tau, Camk2a, α-Synuclein (Snca) and Gap43, which show a dose-dependent rescue, suggesting reduced neuritic pathology upon anti-Aβ treatment (Fig. 2D). To confirm if the lower levels of neuronal markers reflects reduced neuropathology, neurite dystrophy was analysed using Lamp1 immunofluorescence. In concurrence with the reduced number of thiazine^+^ plaques, less Lamp1^+^ neurite dystrophy is observed at 10 mg/kg anti-Aβ (Fig. 2E, F). In addition, we find at 10 mg/kg a significant reduction in the peri-plaque Lamp1 signal (Fig. 2G), suggesting that residual plaques induce less neurite dystrophy in their immediate surrounding. In summary, CSF proteomics detects both neurite pathology and immune cell-related markers, which respond in a dose-dependent manner to anti-Aβ treatment.

### Chronic anti-Aβ treatment partially attenuates DAM activation of microglia without lipid accumulation

To investigate how the observed immune cell-related protein changes in CSF are associated with microglial phenotypes in the brain, bulk 3’-RNA sequencing was performed on Cd11b^+^ MACS-sorted microglia. Microglia sorted from untreated 4-month-old APP-SAA KI mice were included as a baseline/pre-treatment comparison. With aging, in all treatment groups and isotype control, a strong increase in microglial activation is observed, with ~ 4500 differentially expressed genes (DEGs, false discovery rate (FDR) < 5%) (Fig. S5A). These changes correlate to changes identified by Xia et al. 2022, between 8-month-old APP-SAA KI mice compared to WT, validating the 4-month-old time point as a pre-disease control (Fig. S5B). Gene set enrichment analysis (GSEA) confirms an enrichment in gene sets associated with metabolic pathways, including oxidative phosphorylation, glycolysis, MTORC1 signaling, and cholesterol homeostasis, which show predominantly increased gene expression with age (Fig. S5C, D). The gene expression signatures observed are in line with previously defined microglial states in AD [94] showing a strong increase in DAM, interferon (IFN), major histocompatibility complex (MHC), and proliferation-associated gene expression and a reduction in homeostatic gene expression (Fig. S5E), confirming that between 4 and 8 months of age a strong induction of AD-associated microglial signatures is induced in APP-SAA KI mice.

**Fig. 3:**
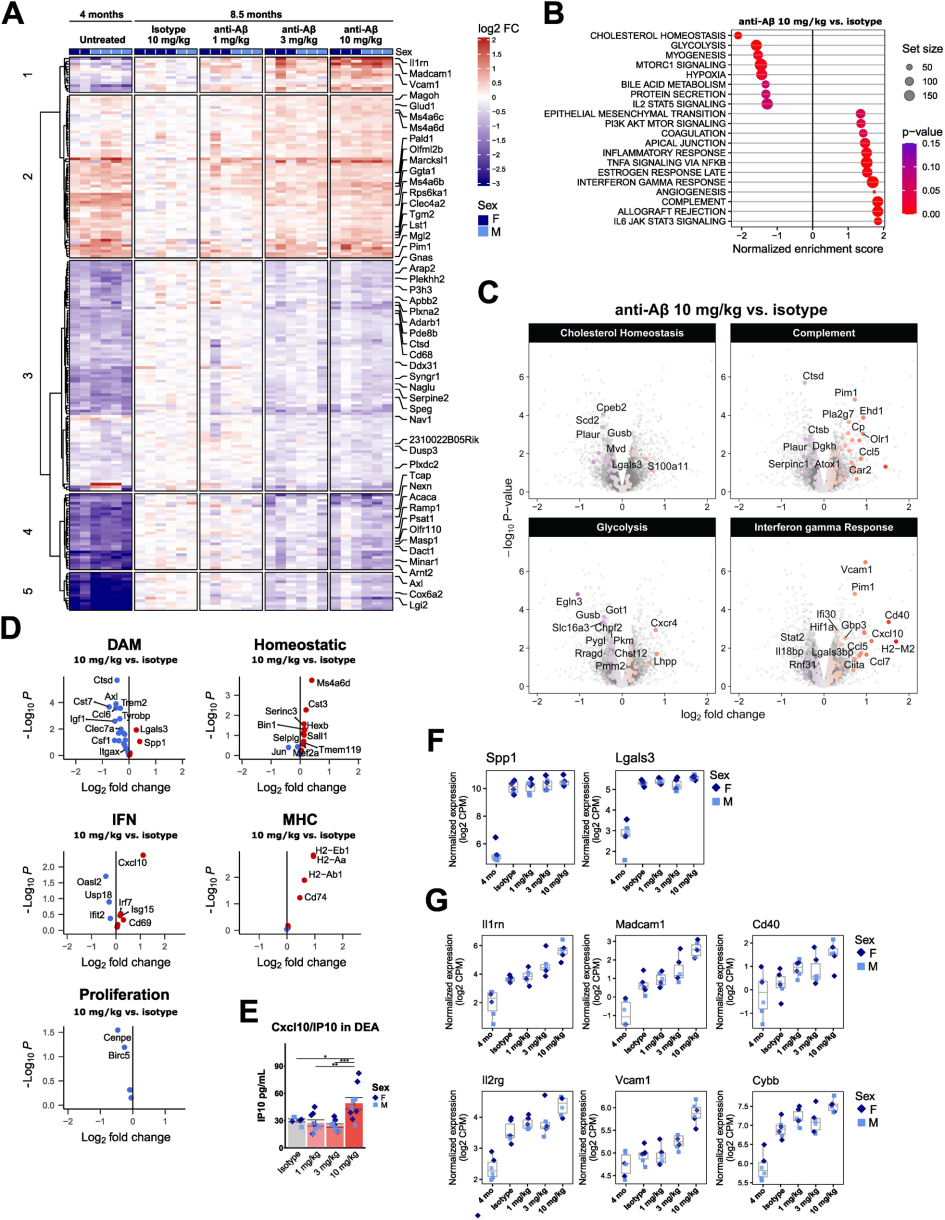
Chronic anti-Aβ treatment partially returns microglia to a pre-disease transcriptional signature. (**A**) Heatmap showing the top 200 differentially expressed genes relative to isotype-treated mice. (**B**) Gene set enrichment analysis (GSEA). (**C**) Volcano plots showing differentially expressed genes related to GSEA pathways. (**D**) Volcano plots showing differentially expressed genes related to microglial states from Chen et al. 2021. (**E**) ELISA quantification of Cxcl10/IP10 protein in DEA fraction. (**F**) Boxplots showing gene expression changes in DAM genes *Spp1* and *Lgals3*. (**G**) Boxplots showing examples of genes that are upregulated upon anti-Aβ treatment. CPM = counts per million. *: *P* < 0.05; **: *P* < 0.01; ***: *P* < 0.001. One-way ANOVA with Tukey’s post hoc test (**E**). For (**A**, **B**, **C**, **D**, **F**, **G**): untreated *n* = 2 f, 4 m, isotype *n* = 4 f, 2 m, 1 mg/kg *n* = 3 f, 3 m, 3 mg/kg *n* = 3 f, 3 m, 10 mg/kg *n* = 3 f, 3 m. For (**E**): isotype *n* = 3 f, 3 m, 1 mg/kg *n* = 5 f, 4 m, 3 mg/kg *n* = 4 f, 5 m, 10 mg/kg *n* = 5 f, 4 m

When comparing anti-Aβ treatment to isotype, polynomial modelling identified a linear association between anti-Aβ dose and gene expression, detecting ~ 400 DEGs (FDR < 5%). Predominantly, a dose-dependent attenuation of genes induced during disease progression is observed with anti-Aβ treatment compared to 4-month-old mice at baseline (Fig. 3A). GSEA reveals an enrichment in genes associated with cholesterol homeostasis and glycolysis, which are predominantly driven by genes that are reduced in expression upon treatment, as well as complement and interferon signaling, which are predominantly driven by genes that are increased in expression compared to isotype (Fig. 3B, C).

When taking a closer look at gene sets associated with known microglial states, we observe attenuated expression of DAM-associated genes such as *Axl*, *Ctsd*, *Cst7*, *Trem2*, *Tyrobp* and *Clec7a*, and increased expression of homeostatic genes, such as *Ms4a6d*, *Cst3*, *Sall1*, *Hexb* and *Tmem119* (Fig. 3D). However, certain MHC- and IFN-associated genes show a further increase upon anti-Aβ treatment, such as *Cxcl10*, of which the encoding protein IP10 is found to be increased in DEA soluble brain extract at 10 mg/kg (Fig. 3E). Interestingly, DAM-associated genes *Lgals3* (encoding galectin-3) and *Spp1* (encoding osteopontin) do not show a dose response to treatment, suggesting that early intervention anti-Aβ treatment does not completely attenuate the full DAM signature (Fig. 3F). The six genes that show the strongest change in expression upon anti-Aβ treatment include *Cd40* (antigen presentation), *Cybb* (Nox2, oxidative burst), *Vcam1* and *Madcam1* (cell adhesion), *Il2rg* and *Il1rn* (cytokine signaling) (Fig. 3G).

**Fig. 4:**
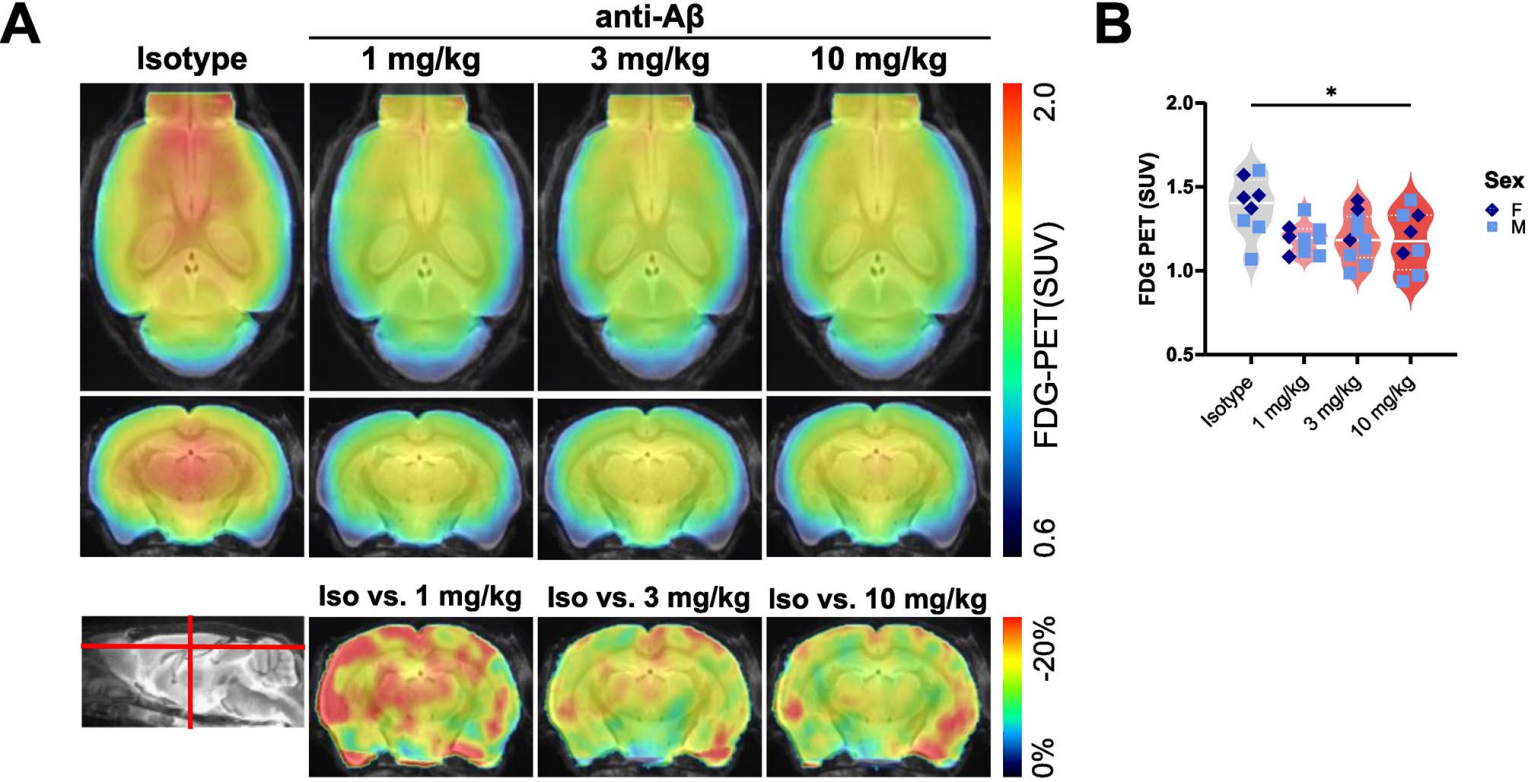
Chronic anti-Aβ treatment decreases microglial glucose uptake. (**A**) Axial and coronal FDG-PET (standardised uptake value (SUV)), and coronal FDG-PET (% change from isotype) per group projected upon a standard magnetic resonance imaging (MRI) T1 atlas. (**B**) Quantification of FDG-PET. *: P < 0.05, One-way ANOVA with Tukey’s post hoc test (**B**). For (**B**): isotype *n* = 4 f, 4 m, 1 mg/kg *n* = 3 f, 7 m, 3 mg/kg *n* = 3 f, 7 m, 10 mg/kg *n* = 3 f, 5 m.

These findings indicate that the gene expression changes following anti-Aβ treatment include a combination of changes linked to reduced amyloid levels (increased homeostatic, reduced DAM), as well as treatment-associated changes related to inflammation. To disentangle the anti-Aβ treatment-induced microglial response from those changes that are related to a difference in age-associated Aβ load, genes were identified that are confidently unchanged in expression (genes with 95% confidence intervals ranging from–20% to +20%) (Fig. S6A). When intersecting confidently unchanged genes between 4- and 8 month-old mice, with confidently changed genes upon anti-Aβ treatment relative to isotype, 45 genes are identified whose expression changes are driven by anti-Aβ treatment and not due to amyloid deposition or aging (11 down, 34 up) (Fig. S6B). Interesting genes include *Lrp1*, *Aip11b*, *Irak1*, *Rab32*, *Ms4a6c* and *Ms4a6d* (Fig. S6C). In addition, we looked at genes that change with aging and are further changed upon chronic anti-Aβ treatment, in the opposite direction as one would expect with lower levels of amyloid deposition. Using this approach, an additional 36 genes are identified, whose expression changes are potentially driven specifically by anti-Aβ treatment (Fig. S6D). Some interesting hits are also genes that show the strongest identified changes upon anti-Aβ treatment such as *Il1rn*, *Madcam1*, *Il2rg* and *Cybb* (Fig. 4G), as well as MHC-II complex genes *H2-Eb1* and *H2-Aa*, and genes related to chemokine signaling such as *Ccl12* and *Cxcr4*. Taken together, this analysis provides an overview of genes whose expression change is likely driven by chronic anti-Aβ treatment rather than an effect of lower plaque load.

To investigate whether alterations in gene expression are associated with changes in microglial lipidome, we performed lipidomic analysis of sorted microglia from the same mice. Changes in lipid abundance are observed in microglia with aging (Fig. S7A, B). Although no FDR-corrected significant changes are detected upon anti-Aβ treatment, an overall attenuation with treatment towards the untreated baseline is observed (Fig.S7C). Most notably, ganglioside GM3 shows a dose-dependent response to anti-Aβ, whereas cholesteryl esters (CE) remains high regardless of treatment (Fig. S7D). Interestingly, GM3 was previously shown to be associated with MX-04^+^ microglia in the APP-SAA KI mouse model, whereas CE levels were observed to be lower in MX-04+ microglia [47] (Fig. S7E). These data suggest that GM3 is increased with aging and Aβ pathology in the APP-SAA KI mouse model, whereas CE is increased with aging, but less associated with Aβ pathology. Taken together, these results indicate that early intervention anti-Aβ treatment does not worsen lipid burden in microglia, but mildly attenuates Aβ-induced lipid changes towards pre-treatment baseline and most predominantly the Aβ pathology-associated lipid GM3.

### Chronic anti-Aβ treatment prevents increased microglial FDG uptake and Trem2 expression but DAM activation around residual plaques remains increased

**Fig. 5:**
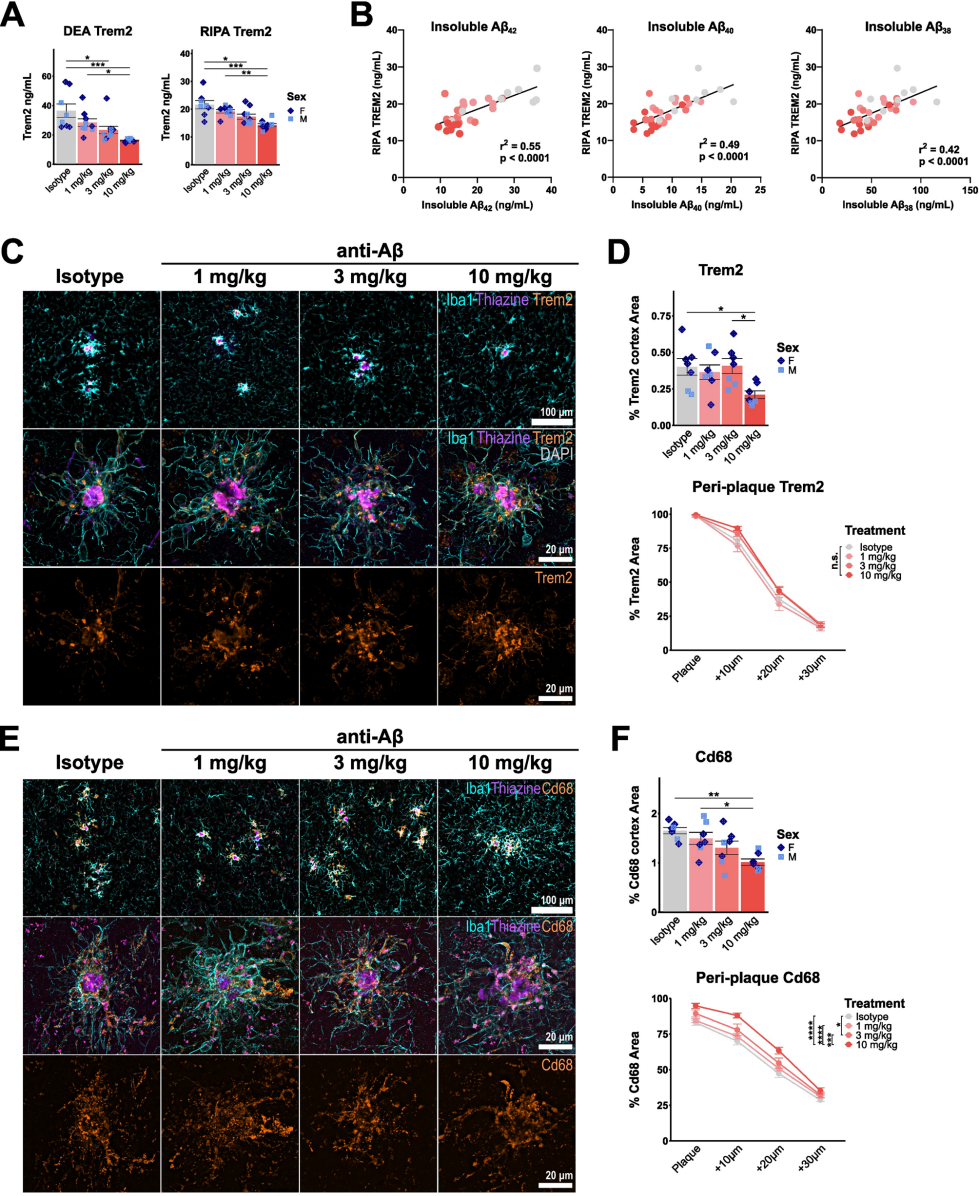
DAM activation is reduced brain-wide, but not around residual plaques. (**A**) ELISA quantification of Trem2 protein in DEA and RIPA brain lysate. (**B**) Correlation between RIPA Trem2 and FA Aβ_42_. (**C**) Representative epifluorescence and confocal images showing DAPI (grey), Iba1 (cyan), thiazine (purple) and Trem2 (orange). (**D**) Quantification of percent cortical and concentric plaque-analysis of Trem2. (**E**) Representative epifluorescence and confocal images showing Iba1 (cyan), thiazine (purple) and Cd68 (orange). (**F**) Quantification of percent cortical and concentric plaque-analysis of Cd68. *: *P* < 0.05; **: *P* < 0.01; ***: *P* < 0.001; ****: *P* < 0.0001. One-way ANOV**A** with Tukey’s post hoc test (**A**, **D**, **F**), Pearson r correlation (**B**). For (**A**, **B**): isotype *n* = 5 f, 3 m, 1 mg/kg *n* = 5 f, 4 m, 3 mg/kg *n* = 5 f, 5 m, 10 mg/kg *n* = 5 f, 4 m. For (**D**, **F**): isotype *n* = 5 f, 2 m, 1 mg/kg *n* = 4 f, 3 m, 3 mg/kg *n* = 4 f, 3 m, 10 mg/kg *n* = 4 f, 3 m

Bulk RNA-seq identified a reduction in overall microglial DAM activation and glycolysis with anti-Aβ treatment. Microglial activation in response to Aβ has previously been shown to correlate with a significant increase in glucose uptake, which is driven by Trem2 expression and further increased upon Trem2 agonism [49, 54, 95, 96]. Based on cell-sorting experiments in mouse models of amyloid pathology, [^18^F]-fluorodeoxyglucose (FDG) PET can be used as a readout of microglial glycolytic activity [54]. To confirm whether the reduced expression of glycolysis-associated genes upon anti-Aβ treatment translates to reduced microglial glucose uptake, we measured FDG-PET after 16 weeks of antibody treatment. Similar to FBB-PET, a reduction in FDG uptake is observed (Fig. 4A, B). In parallel, we find a dose-dependent reduction in brain Trem2 protein levels in both DEA and RIPA brain extracts (Fig. 5A). Trem2 levels correlate with insoluble Aβ_42_, Aβ_40_, and Aβ_38_ levels (Fig. 5B), confirming that the Trem2-induced microglial response is closely linked to the degree of Aβ accumulation. However, as the formation of plaques is not completely prevented with anti-Aβ treatment, we were interested to see whether microglial DAM activation is still induced at residual plaques. Immunostaining confirms reduced total cortical Trem2 and Cd68 area at 10 mg/kg (Fig. 5C - F). Using concentric plaque analysis, we find that treatment did not further increase Trem2 around plaques, but at 10 mg/kg did increase Cd68 (Fig. 5D, F). ApoE follows a similar pattern, with reduced total cortical area at 10 mg/kg, but is not significantly changed in the plaque ‘penumbra’ (Fig. S8A, B). In contrast, homeostatic marker P2ry12 is not reduced in total cortex coverage, but also not around plaques (Fig. S8C, D). Taken together, these findings suggest that microglia at residual plaques do not lose their Aβ-induced DAM activation and appear to still be actively dealing with residual plaques after 16 weeks of early intervention anti-Aβ treatment.

### Chronic anti-Aβ treatment increases microglial clustering and an antibody-induced microglial phenotype around residual plaques

**Fig. 6:**
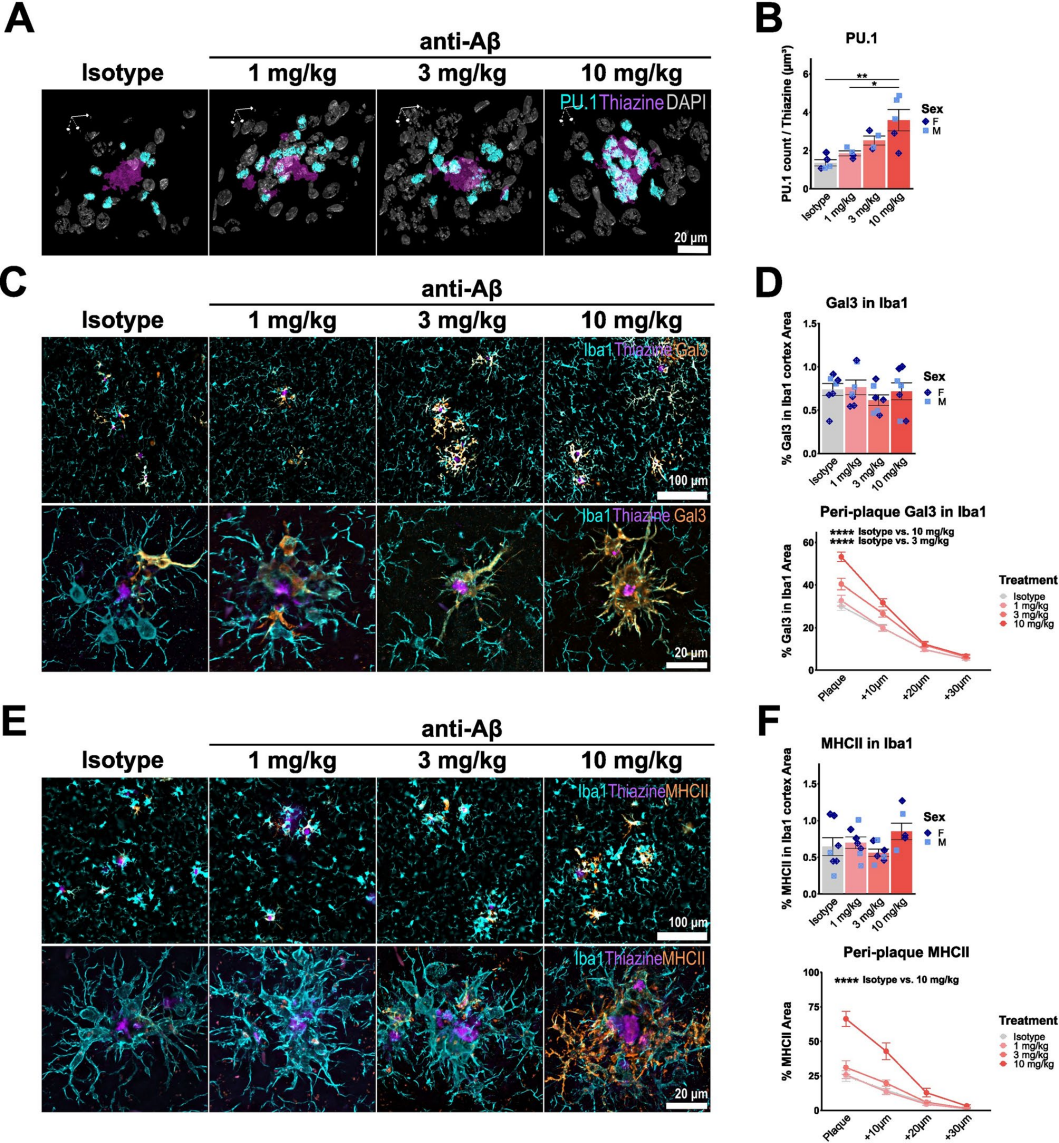
Increased microglial clustering is associated with increased anti-Aβ-induced MHC-II and Galectin-3 around residual plaques. (**A**, **B**) Isotropic 3D rendering of confocal immunofluorescent images showing thiazine (purple), DAPI (grey) and PU.1 (cyan) and quantification of PU.1^+^ DAPI^+^ nuclei number to normalised by plaque volume (**C**) Representative epifluorescence and confocal images showing Iba1 (cyan), thiazine (purple) and co-staining with Galectin-3 (orange). (**D**) Quantification of Galectin-3 in Iba1 in total cortex and in concentric rings around thiazine plaques (**E**) Representative epifluorescence and confocal images showing Iba1 (cyan), thiazine (purple) and co-staining with MHC-II (orange). (**F**) Quantification of MHC-II in Iba1 in total cortex and in concentric rings around thiazine plaques. *: *P* < 0.05; ***: *P* < 0.001; ****: *P* < 0.0001, One-way ANOVA with Tukey’s post hoc test (**B**, **D**, **F**). For (**B**): isotype *n* = 3 f, 2 m, 1 mg/kg *n* = 2 f, 2 m, 3 mg/kg *n* = 2 f, 2 m, 10 mg/kg *n* = 2 f, 3 m. For (**D**, **F**): isotype *n* = 5 f, 2 m, 1 mg/kg *n* = 4 f, 3 m, 3 mg/kg *n* = 4 f, 3 m, 10 mg/kg *n* = 4 f, 3 m

Previous studies have consistently reported increased microglial clustering around plaques upon anti-Aβ treatment in mice and patients [29, 31, 41]. However, using concentric plaque analysis we find no changes in peri-plaque Iba1 (Fig. S9A). It is possible that due to the thickness of the brain sections (50 μm), we are unable to distinguish individual microglia based on Iba1 signal with epifluorescence. Therefore, we employed a more sensitive analysis by performing confocal imaging of entire plaques and co-stained with PU.1, a microglial-specific transcription factor. Using 3D reconstruction of PU.1^+^ nuclei and plaques, we can confirm a dose-dependent increase in microglia localised with cell bodies in close proximity (within 10 μm) to plaques (Fig. 6A, B). In addition to microglia, it was recently suggested that acute anti-Aβ treatment can increase astrogliosis, with a trend towards increased Gfap around plaques [29]. In contrast to these findings, we find no changes in total cortex area coverage of astrogliosis marker Gfap after chronic anti-Aβ treatment, however we do observe increased Gfap coverage at 10 mg/kg around residual plaques (Fig. S9B, C), suggesting that treatment can potentially induce both peri-plaque microgliosis and astrogliosis.

To further investigate antibody-treatment induced effects specifically around residual plaques, we stained for Galectin-3 (Fig. 6C, D) and MHC-II (Fig. 6E, F), which we showed to be increased in expression in microglia after anti-Aβ treatment. Despite the finding that percent Galectin-3 and MHC-II coverage in microglia is not different between treatment groups in the cortex using immunofluorescence, a clear dose-dependent increase in protein levels is observed in concentric rings around amyloid plaques for both proteins (Fig. 6D, F). Taken together, these findings indicate increased microglial clustering around plaques, with the concomitant induction of a specific peri-plaque microglial state that is associated with antigen presentation, phagocytosis, and microglial recruitment to plaques.

## Discussion

**Fig. 7:**
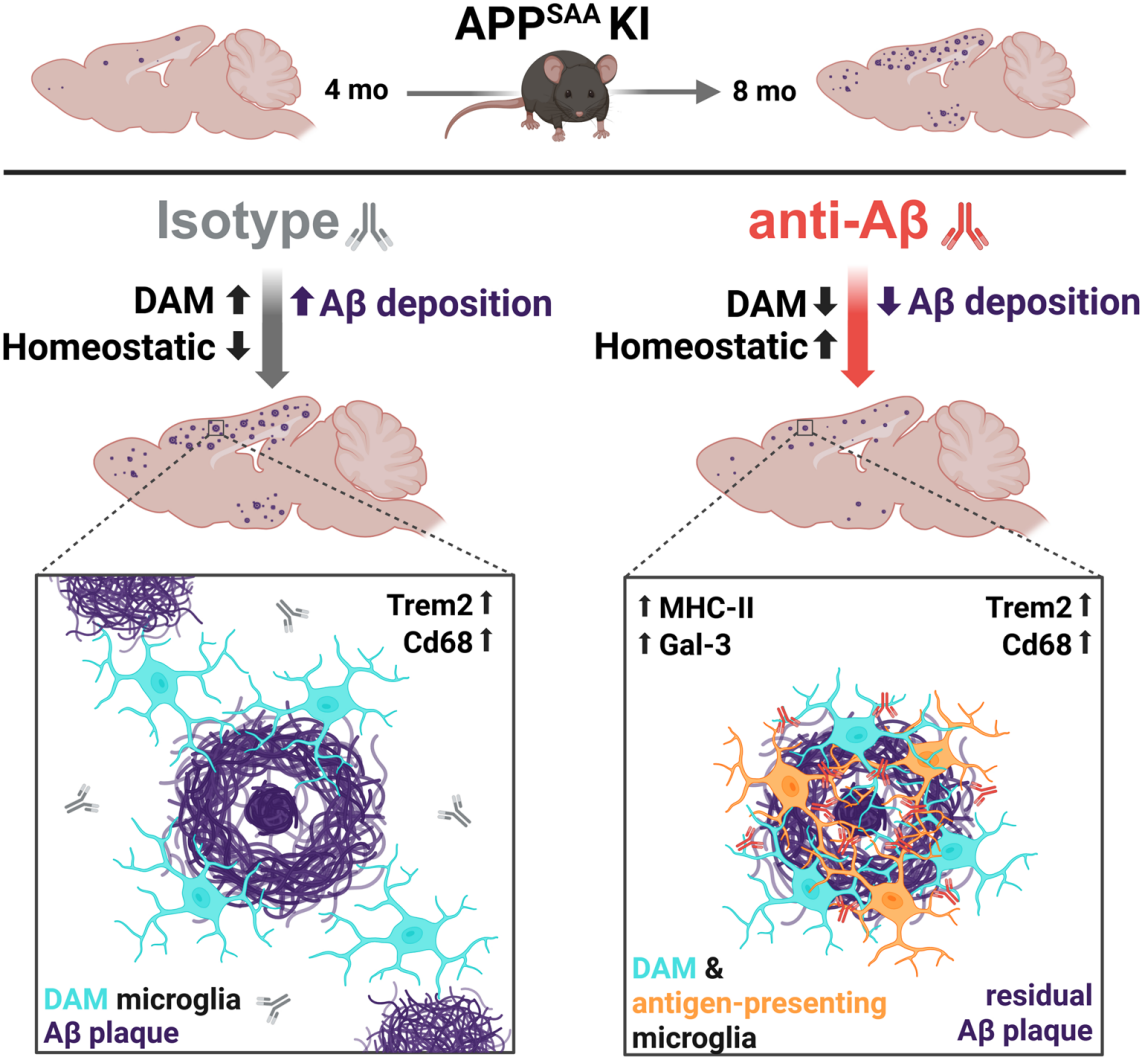
Graphical overview of brain-wide effects, as well as microglial response at residual plaques, upon early intervention chronic anti-Aβ antibody treatment in APP-SAA KI mice

The current study suggests that anti-Aβ treatment results in a dose-dependent removal of aggregated Aβ and reduced plaque formation in APP-SAA KI mice upon chronic treatment. Concomitantly, microglial DAM activation is decreased in a manner that correlates with the reduction in plaque load. However, microglial clustering around residual plaques is increased and these cells display a unique antibody-driven microglial phenotype (Fig. 7).

The dose-dependent reduction of amyloid observed in APP-SAA KI mice is in line with the seminal work from Sevigny et al. 2016, who reported a dose-dependent removal of amyloid plaques in mice and patients [31]. Aducanumab is an antibody that has a strong preference for Aβ fibrils over protofibrils and monomers and should therefore be most efficient at removing aggregated Aβ species [97, 98]. Indeed, plaques in the APP-SAA KI mouse contain relatively small dense cores and a large amount of diffuse fibrillar Aβ, which was more efficiently removed by chronic anti-Aβ treatment. However, despite starting at an early disease time-point where only few plaques should be present, dense-core plaque formation still occurred and was only significantly reduced at the highest treatment dose. Similar findings were reported by previous studies, showing that chronic anti-Aβ treatment does not effectively remove dense-core plaques, but removes small and diffuse Aβ deposits and inhibits progression of plaque deposition [8, 28, 33, 37, 40]. These results appear to contradict clinical trial data, which report a strong removal of amyloid (59–71% decrease in PET) after chronic Aducanumab treatment in patients [4, 41]. Whether these discrepancies are due to differences in treatment paradigms, differences in effector function due to chimerisation of the antibody, or an inherent difference in the immune cell response between organisms remains to be determined. Interestingly however, a post-mortem case study of a patient treated with 32 monthly doses of Aducanumab, as well as cases of active anti-Aβ immunisation, described sparsely persisting dense-core plaques after treatment, which lacked or showed ‘moth-eaten’ diffuse halos, suggesting a more efficient removal of diffuse fibrils over dense-core plaques even in patients [41]. Taken together, these findings indicate that anti-Aβ treatment could be most effective at preventing plaque formation at early disease stages where amyloid is less densely aggregated.

In patients, anti-Aβ antibody treatment is associated with an increased risk of oedema (ARIA-E) and haemorrhages (ARIA-H) that could potentially be lethal in rare cases [99, 100]. Several mechanisms for these side effects have been postulated, including the shifting of Aβ from the parenchyma to the blood vessels and the immune response of microglia or perivascular macrophages [11, 12, 40]. The APP-SAA KI mouse model used in this study displayed Prussian blue deposits with antibody treatment, but also in an age-dependent manner in untreated mice. Whether this phenotype could be related to the hTfR KI [48] in these mice is unclear, however another study using 5xFAD; hTfR KI mice did not observe any spontaneous microbleeds without anti-Aβ antibody treatment [44]. We found no significant increase in Prussian Blue deposits with anti-Aβ over isotype antibody treatment, although more sensitive methods would be required to fully capture the extent of microbleeds upon treatment.

Proteomic analysis of terminally-collected CSF indicated a marked reduction in total CSF CamKIIα, Tau and α-Syn almost to baseline (untreated 3 months of age) levels, despite the remaining presence of some amyloid plaques and Lamp1^+^ neuritic dystrophy. In clinical trials, CSF total Tau levels were reduced after treatment with Aducanumab and other Aβ-targeting antibodies [4–6]. No data on CSF total α-Syn levels after anti-Aβ antibody treatment have been previously reported, but increased CSF total α-Syn was previously found to be associated with increased risk of cognitive decline in non-demented adults [101] and reported to be increased in patients diagnosed with probable AD [102]. Unlike patients, APP-SAA KI mice do not develop Tau tangles and neurodegeneration, nor do they develop co-pathologies such as Lewy bodies. It is therefore likely that increased CSF α-Syn, Tau, CamKIIa and Gap-43 upon aging in APP-SAA KI mice reflect neuritic degeneration, rather than neuronal loss or a Lewy-body like co-pathology [103]. Interestingly, two previous studies of anti-Aβ treatment in models of amyloid deposition in combination with human Tau overexpression, reported less Tau phosphorylation and axonal swellings or less Tau^+^ dystrophic neurites around plaques [33, 37] hinting at reduced Aβ neurotoxicity in the plaque penumbra. In line with these findings, we observed a decrease in Lamp1^+^ neurite dystrophy around residual plaques with 10 mg/kg anti-Aβ treatment. It is possible that increased microglial clustering around plaques upon anti-Aβ treatment, which is consistently reported in previous studies [29, 31, 33, 36, 37, 41–45] and confirmed in the current study, could play a role by forming a protective barrier against the release of synaptotoxic Aβ fibrils or oligomers, but this hypothesis warrants further investigation [37, 104–106].

CSF proteomics also indicated microglial changes upon anti-Aβ treatment. To investigate the effect of anti-Aβ treatment on microglial states and phenotypes in more detail, we performed bulk RNA-sequencing of isolated Cd11b^+^ microglia. We identified a relatively small, but dose-dependent effect on gene expression after chronic anti-Aβ treatment that largely showed an attenuation of the age- and pathology-associated gene expression changes in the APP-SAA KI model. Gene expression related to the DAM signature was reduced and homeostatic gene expression was increased. Furthermore, gene set enrichment analysis (GSEA) indicated an attenuation of gene expression associated with metabolic pathways such as cholesterol homeostasis and glycolysis.

In agreement with these findings, LC-MS analysis of microglial lipid composition showed a mild reversal of the age-induced lipid changes in APP-SAA KI mice. Ganglioside GM3 levels displayed a dose-dependent reduction upon anti-Aβ treatment. GM3 was previously found to be increased with amyloid pathology in APP-SAA KI mice and is enriched in microglia that have phagocytosed MX-04^+^ Aβ [47]. In addition, lowering of GM3 was previously found to be associated with an attenuation of amyloid pathology [107]. Overall, lipidome analysis suggests that microglia do not show increased lipid burden upon chronic early intervention anti-Aβ treatment, but rather an attenuation towards pre-treatment baseline.

A reduction in microglial glycolysis was corroborated by FDG-PET, showing a decrease in cortical glucose uptake. In addition, a reduction in brain Trem2 and sTrem2 protein levels correlated with insoluble Aβ_38,_ Aβ_40_, and Aβ_42_, suggesting that the observed reduction in DAM activation is mainly associated with reduced plaque deposition after chronic treatment. Recently, a similar association was reported for brain Trem2 and Aβ_38_, but not Aβ_40_ and Aβ_42_ [108]. In addition, we observed a sex-specific effect on Trem2 protein levels in CSF, but not in brain, although this has to be interpreted with caution as these measurements were derived from two separate cohorts. Interestingly, previous studies that reported reduced microglial DAM activation and/or increased homeostatic gene expression after anti-Aβ treatment in mice after various dosing regimens at later stages of pathology, did so despite observing no to very little removal of Aβ [29, 30, 34]. It is possible that changes in Aβ levels may have been underestimated in these studies or that Fc gamma receptor (FcγR)-mediated Syk signaling directly modulates microglial DAM activation, though this remains to be investigated. 

Although gene expression changes overall showed an attenuation towards pre-treatment baseline, we found that anti-Aβ treatment did not lower expression of the DAM genes *Lgals3* and *Spp1.* Moreover, a specific set of genes were strongly increased by anti-Aβ treatment in a dose-dependent manner. These genes are associated with antigen presentation (*Cd40*, *H2-Eb1*, *H2-Aa*), cell adhesion (*Madcam1*, *Vcam1*), ROS production (*Cybb*) and cytokine/chemokine signaling (*Il2rg*, *Il1rn, Ccl12, Cxcr4*). A subset of these we were able to associate with anti-Aβ treatment, independent of differences in age-associated amyloid deposition, including decreased expression of *Lrp1* and increased expression of *Msa46c* and *Ms4a6d*. Increased MHC-II-associated gene expression upon anti-Aβ treatment was also observed in two previous reports of chronic Aducanumab treatment [29, 34]. Interestingly, Il-33-induced *Vcam1* and *MHC-II* expression in microglia was previously shown to play a crucial role in chemotaxis towards plaques and subsequently promote plaque removal [109] suggesting this response could be protective. Moreover, *Cybb* and MHC-II expression were previously identified as markers of microglia with low lipid droplet burden [110] indicating that amyloid clearance by microglia upon anti-Aβ treatment is likely not associated with lipid droplet accumulation in the current paradigm. Another interesting hit is the increase in *Il1rn*, encoding interleukin 1 receptor antagonist, a negative regulator of innate immune cell activation [111, 112]. *Il1rn* has previously been shown to be associated with inhibition of NLRP3 inflammasome activation and reducing the vascular inflammatory effects of IL1β [113]. Interestingly, increased IL1RN levels have also been found after TREM2 agonistic antibody treatment in mice and patients [114, 115]. It is possible that the increased expression of *Cxcl10, Ccl12, Cxcr4* and *Il1rn* modulates T-cell recruitment and activation [116], but whether significant T-cell infiltration occurs upon anti-Aβ antibody treatment and whether this relates to treatment efficacy remains to be determined.

To validate our sequencing results and understand how these changes in microglial gene expression relate to the presence of residual plaques after early intervention chronic anti-Aβ treatment, we performed immunofluorescent staining and found an increase of markers Cd68, Galectin-3 and MHC-II specifically around plaques, whereas Trem2 was induced around plaques similarly across treatment groups. Taken together these results suggest that microglia that cluster around residual plaques maintain their DAM signature, but also acquire an antigen presentation phenotype in a dose-dependent manner. Whether this phenotype is beneficial or detrimental to downstream pathology remains to be determined. A previous study suggested that upon treatment cessation and washout, microglia display blunted DAM activation, but this finding may be confounded by an increase in DAM activation in isotype control-treated animals over time [29]. Therefore, in follow-up studies it would be important to further investigate microglial responses, plaque engagement, and the effects on disease progression after treatment cessation.

The current study has several limitations. Firstly, the APP-SAA KI mouse model does not display Tau pathology, neurodegeneration, or overt behavioural phenotypes related to memory function at 8 months of age [47] and can therefore not be used to draw conclusions about the potential consequences of chronic amyloid removal on parameters that would be relevant in the clinic. Secondly, it is important to note that the current treatment paradigm investigates an early intervention as opposed to most human trials that start treatment in patients who are already amyloid PET-positive and have some memory decline and is therefore less comparable to findings from the clinic, although prevention studies are also under investigation for familial early-onset AD [117]. In addition, at the age of 4.5 months APP-SAA mice likely do not have CAA-like amyloid deposits, therefore this paradigm might not be suitable for the detection of chronic anti-Aβ antibody treatment effect on microbleeds. Thirdly, although some sex-specific immune response effects were observed, the current study was underpowered to make claims about sex-specific treatment effects, which have been reported previously [29]. Lastly, by opting to perform bulk microglia transcriptomic analysis, we cannot identify specific microglia sub-populations that respond to anti-Aβ antibody treatment. Although we correlate the occurrence of certain transcriptomic phenotypes as localised around plaques based on Galectin-3 and MHC-II immunofluorescent staining, spatial transcriptomic analysis would be required to unbiasedly characterise expression of these genes in relation to plaques.

Passive immunotherapy approaches have managed to bypass many of the side effects of active immunization, but little is known about the long-term effects of this treatment on microglia, despite being key players in the efficient removal of Aβ. As anti-Aβ antibodies are administered chronically to an increasing number of patients, it is essential to better understand the effect of such treatments on the immune system. We find that overall microglial activation is reduced in correlation to plaque load, but microglia at residual plaques acquire a unique phenotype of both DAM and a select set of genes associated with antigen presentation. The finding that Trem2 is still induced around residual plaques after early intervention chronic treatment indicates that microglia are capable of actively dealing with Aβ on the long term and suggests that continued dosing with an anti-Aβ antibody could be a valid long-term treatment option, provided that treatment is given at an early time point in amyloid disease stage. Moreover, potential (co-)treatment with an agonistic Trem2 antibody might also be most effective during the earliest disease stages [23]. In summary, our findings highlight the importance of early intervention with anti-Aβ antibodies and support the therapeutic potential of combining such treatment with strategies that enhance microglial function, to optimise long-term outcomes in AD.

## Electronic Supplementary Material

Below is the link to the electronic supplementary material.

Supplementary Figures

Uncropped Blots

Supplementary Figure 1

Supplementary Figure 2

Supplementary Figure 3

Supplementary Figure 4

Supplementary Figure 5

Supplementary Figure 6

Supplementary Figure 7

Supplementary Figure 8

Supplementary Figure 9

## Data Availability

LC-MS/MS proteomics data have been deposited to the ProteomeXchange Consortium via the PRIDE partner repository with the dataset identifier PXD061932. Bulk RNA-seq data have been deposited to the Gene Expression Omnibus (GEO) repository with accension number GSE288801. Data analysis scripts for reproducible analysis of bulk RNA-seq data have been deposited on Zenodo under digital object identifier https://doi.org/10.5281/zenodo.14812455.

## Abbreviations

α-Syn: α-Synuclein
Aβ: Amyloid β-peptide
AD: Alzheimer’s disease
APP: Amyloid precursor protein
ARIA: Amyloid-related imaging abnormalities
ARIA-E: ARIA-related oedema
ARIA-H: ARIA-related haemorrhage
BCA: Bicinchoninic acid
BSA: Bovine serum albumin
CAA: Cerebral amyloid angiopathy
CamKIIα: Calcium/calmodulin-dependent protein kinase type II subunit alpha
CE: Cholesteryl esters
COA: Cortico-amygdala area
CSF: Cerebrospinal fluid
CTF: C-terminal fragment
DAM: Disease-associated microglia
DAPI: 40,6-diamidino-2-phenylindole
DEA: Diethylamine
DEG: Differentially expressed gene
diaPASEF: Data-independent acquisition parallel accumulation-serial fragmentation
ELISA: Enzyme-linked immunosorbent assay
EtOH: Ethanol
FA: Formic acid
FcγR: Fc gamma receptor
FBB: Florbetaben
FDG: Fluorodeoxyglucose
FDR: False discovery rate
Gfap: Glial fibrillary acidic protein
GM3: Monosialodihexosylganglioside 3
GSEA: Gene set enrichment analysis
HBSS: Hanks’ buffered salt solution
hTfR: Human transferrin receptor
IFN: Interferon
Il1rn: Interleukin-1 receptor antagonist
i.p.: Intraperitoneal
KI: Knock-in
Lamp1: Lysosomal-associated membrane protein 1
LC-MS: Liquid chromatography-mass spectrometry
LC-MS/MS: Liquid chromatography-tandem mass spectrometry
LOAD: Late-onset Alzheimer’s disease
MACS: Magnetic-activated cell sorting
MBq: Megabecquerel
MCI: Mild cognitive impairment
MHC: Major histocompatibility complex
MMF: Medetomidine-midazolam-fentanyl
MR: Magnetic resonance
MRI: Magnetic resonance imaging
MSD: Meso scale discovery
MX-04: Methoxy-04
NaCl: Sodium chloride
NDS: Normal donkey serum
NLRP3: NOD-, LRR-, and pyrin domain-containing protein 3
PBS: Phosphate-buffered saline
PET: Positron emission tomography
PFA: Paraformaldehyde
RIPA: Radioimmunoprecipitation assay
RNA-seq: RNA-sequencing
ROI: Region of interest
ROS: Reactive oxygen species
RT: Room temperature
SEM: Standard error of the mean
SUV: Standard uptake value
TBS: Tris-buffered saline
TIMS: Trapped ion mobility spectrometry
Trem2: Triggering receptor expressed on myeloid cells 2
VOI: Voxel of interest
VT: Total volume of distribution
