# Supplementary material for: Early intervention anti-Aβ immunotherapy attenuates microglial activation without inducing exhaustion at residual plaques
Source: Mol Neurodegener. 2025 Aug 20;20:92. doi: 10.1186/s13024-025-00878-1 (PMC12366171; doi:10.1186/s13024-025-00878-1)
Supplement: Supplementary file 1 — Supplementary Figures [file 13024_2025_878_MOESM1_ESM.docx]

**Supplementary figures to**

**Early intervention anti-Aβ immunotherapy attenuates microglial activation without inducing exhaustion at residual plaques**

Lis de Weerd^1,#^, Selina Hummel^2^, Stephan A. Müller^1,7^, Iñaki Paris^1,§^, Thomas Sandmann^3^, Marie Eichholtz^6^, Robin Gröger^2^, Amelie L. Englert^2^, Stephan Wagner^2^, Connie Ha^3^, Sonnet S. Davis^3^, Valerie Warkins^3^, Dan Xia^3^, Brigitte Nuscher^4^, Anna Berghofer^1^, Marvin Reich^1,†^, Astrid F. Feiten^4^, Kai Schlepckow^1^, Michael Willem^4^, Stefan F. Lichtenthaler^1,5,7^, Joseph W. Lewcock^3^, Kathryn M. Monroe^3^, Matthias Brendel^1,2,5^, Christian Haass^1,4,5,#^

^1^ German Center for Neurodegenerative Diseases (DZNE), Munich, Germany.

^2^ Department of Nuclear Medicine, University Hospital of Munich, Ludwig Maximilians University, Munich, Germany.

^3^ Denali Therapeutics, Inc., South San Francisco, USA.

^4^ Metabolic Biochemistry, Biomedical Center, Faculty of Medicine, Ludwig-Maximilians Universität (LMU), Munich, Germany.

^5^ Munich Cluster for Systems Neurology (SyNergy), Munich, Germany.

^6^ Biochemistry Master’s Program, Gene Center Munichs, Ludwig-Maximilians University (LMU), Munich, Germany.

^7^ Neuroproteomics, School of Medicine and Health, Klinikum Rechts der Isar, Technical University of Munich (TUM), Munich, Germany.

^†^ Current address: Department of Neurology and Neurological Sciences, Stanford University School of Medicine, Stanford University, Stanford, CA, USA

^#^ Corresponding authors:

Christian Haass, PhD
Email: [christian.haass@mail03.med.uni-muenchen.de](mailto:christian.haass@mail03.med.uni-muenchen.de)

Lis de Weerd
Email: [lis.deweerd@dzne.de](mailto:lis.deweerd@dzne.de)


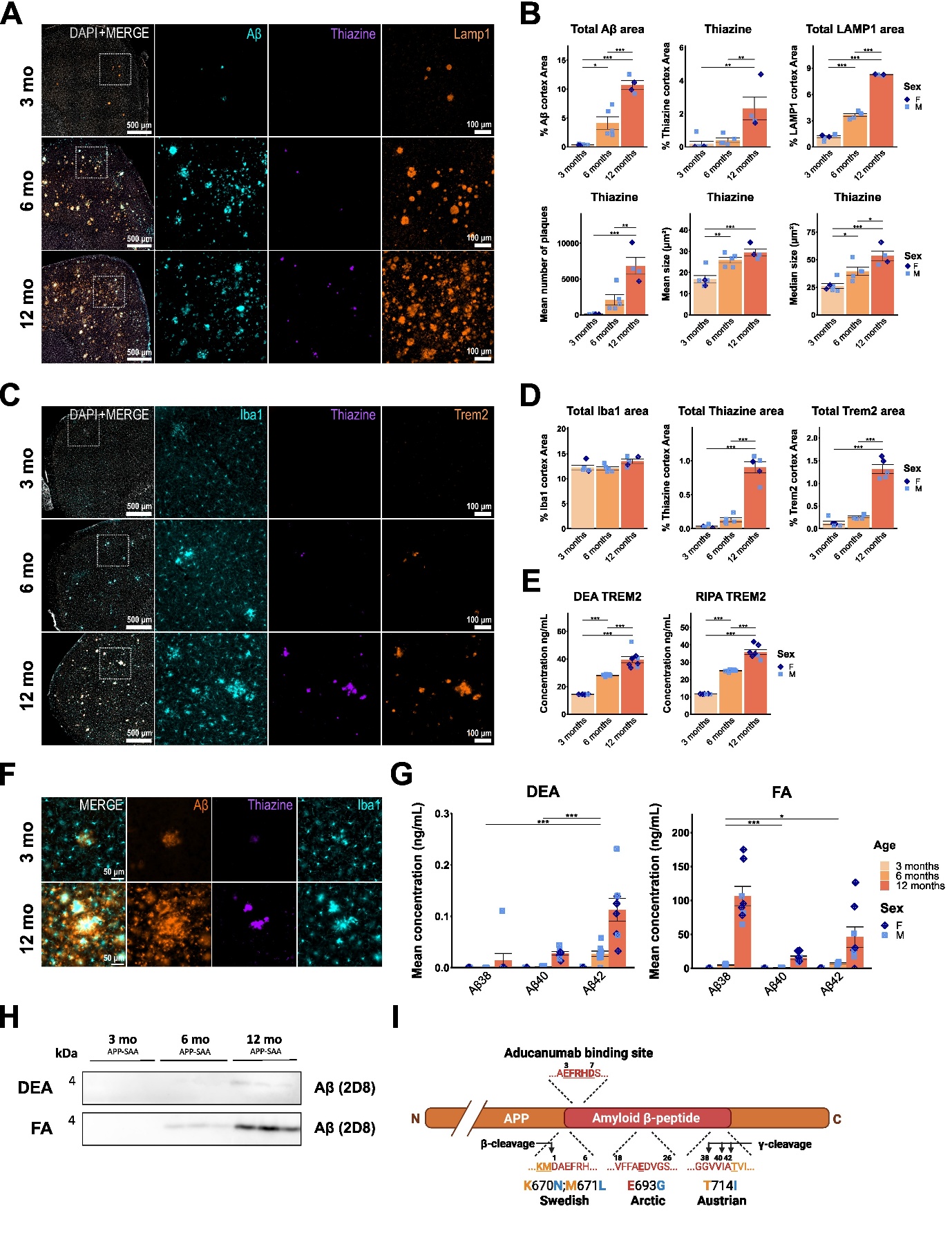


**Figure S1:** **Progression of amyloid pathology in the APP-SAA mouse model**

(**A**) Representative immunofluorescent images of sagittal cortical sections showing Aβ (cyan), thiazine (purple), and Lamp1 (orange) and (**B**) quantification of percent cortical thiazine area, plaque count and size, and percent cortical Lamp1 area. (**C**) Representative immunofluorescent images of sagittal cortical sections showing Iba1 (cyan), thiazine (purple) and Trem2 (orange) and (**D**) quantification of percent cortical area of Iba1, thiazine and Trem2. (**E**) ELISA quantification of Trem2 in DEA and RIPA brain lysate. (**F**) Representative immunofluorescent images showing Aβ (orange), thiazine (purple), and Iba1 (cyan), and microglia clustering around diffuse Aβ plaques at 3 months and a comparison to dense plaques at 12 months. (**G**) Aβ-triplex ELISA quantification of DEA- and FA-extracted Aβ. (**H**) Western blot of DEA and FA fractions for Aβ (antibody 2D8). (**I**) Schematic of the human APP protein with Swedish, Arctic, and Austrian mutations and anti-Aβ (Aducanumab) binding site indicated. *: P < 0.05; **: P < 0.01; ***: P < 0.001. One-way ANOVA with Tukey’s post hoc test (B, D). Two-way ANOVA with Tukey’s post hoc test (G). Schematic (F) was created with BioRender.com. For (B): 3 months *n* = 2 f, 3 m, 6 months *n* = 5 m, 12 months *n* = 2 f, 2 m. For (D): 3 months *n* = 2 f, 3 m, 6 months *n* = 5 m, 12 months *n* = 2 f, 3 m. For (E, G): 3 months *n* = 4 f, 3 m, 6 months *n* = 9 m, 12 months *n* = 5 f, 3 m.


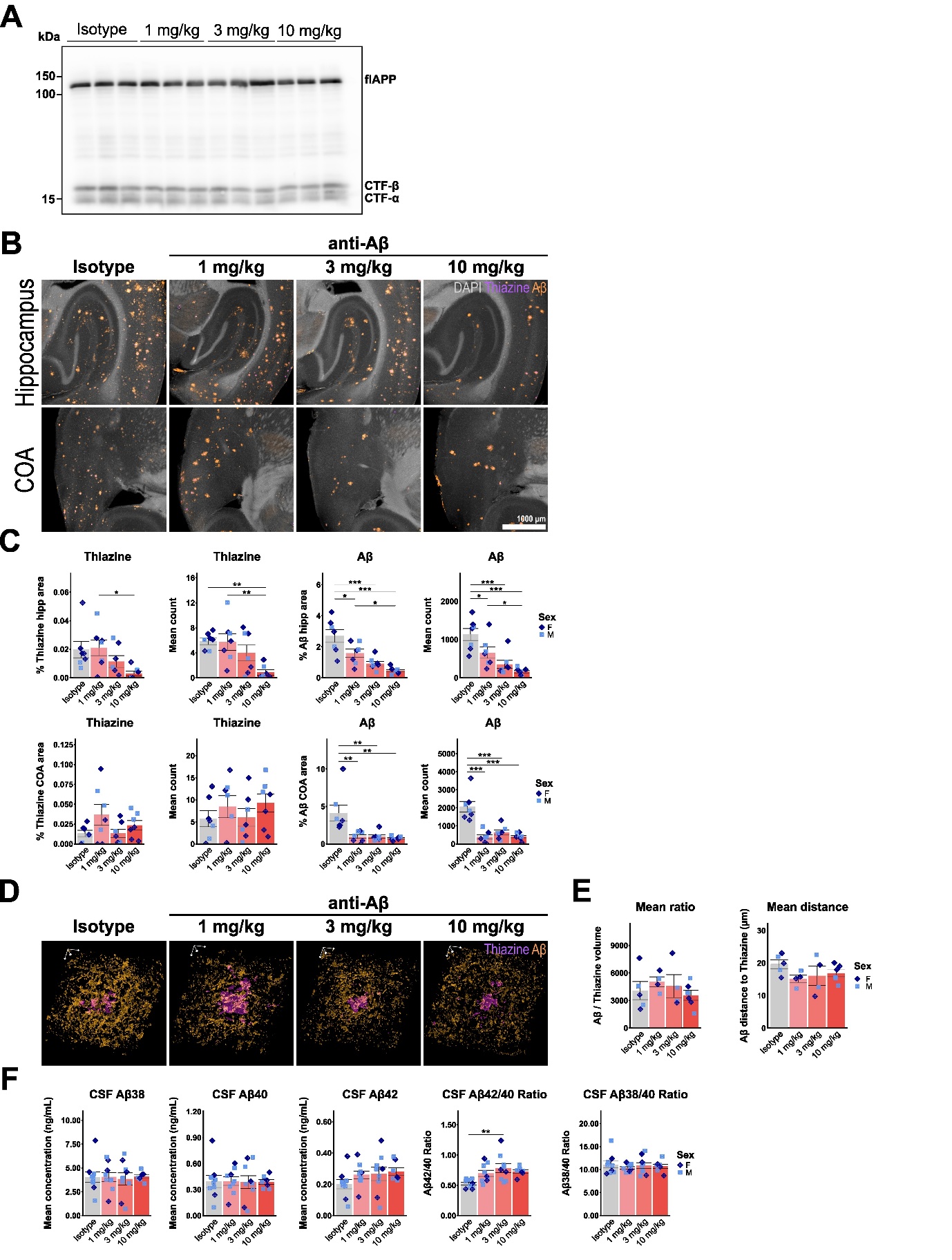


**Figure S2: Chronic anti-Aβ treatment reduces amyloid-β levels in a dose-dependent manner**(**A**) Western blot showing full-length APP, CTF-β, and CTF-α in RIPA brain extract. (**B**) Representative immunofluorescent images of sagittal hippocampus and cortico-amygdala area (COA) sections showing DAPI (grey), thiazine (purple), and Aβ (3552 antibody, orange). (**C**) Quantification of percent cortical thiazine and Aβ area and number in the hippocampus and COA. (**D**) Isotropic 3D rendering of confocal immunofluorescent images showing thiazine (purple) and Aβ (3552 antibody, orange). Scale bar = 10 µm. (**E**) Quantification of mean Aβ/thiazine ratio and mean distance of Aβ to thiazine border. (**F**) ELISA quantification of Aβ_38_, Aβ_40_ and Aβ_42_, as well as the Aβ_42/40_ and Aβ_38/40_ ratio in terminally-collected CSF. *: P < 0.05; **: P < 0.01; ***: P < 0.001. One-way ANOVA with Tukey’s post hoc test (C, E, F). For (C): isotype *n* = 5 f, 3 m, 1 mg/kg *n* = 5 f, 4 m, 3 mg/kg *n* = 5 f, 5 m, 10 mg/kg *n* = 5 f, 4 m (same samples as Fig. 1F, 2F, 5D, F, 6D, F). For (E): isotype *n* = 3 f, 2m, 1 mg/kg *n* = 3 f, 2 m, 3 mg/kg *n* = 3 f, 2 m, 10 mg/kg *n* = 2 f, 3 m (same samples as Fig. 6A, B). For (F): isotype *n* = 3 f, 6 m, 1 mg/kg *n* = 3 f, 6 m, 3 mg/kg *n* = 3 f, 6 m, 10 mg/kg *n* = 3 f, 4 m (same samples as Fig. 2A, B, C, Fig. S4A, B, C).

**
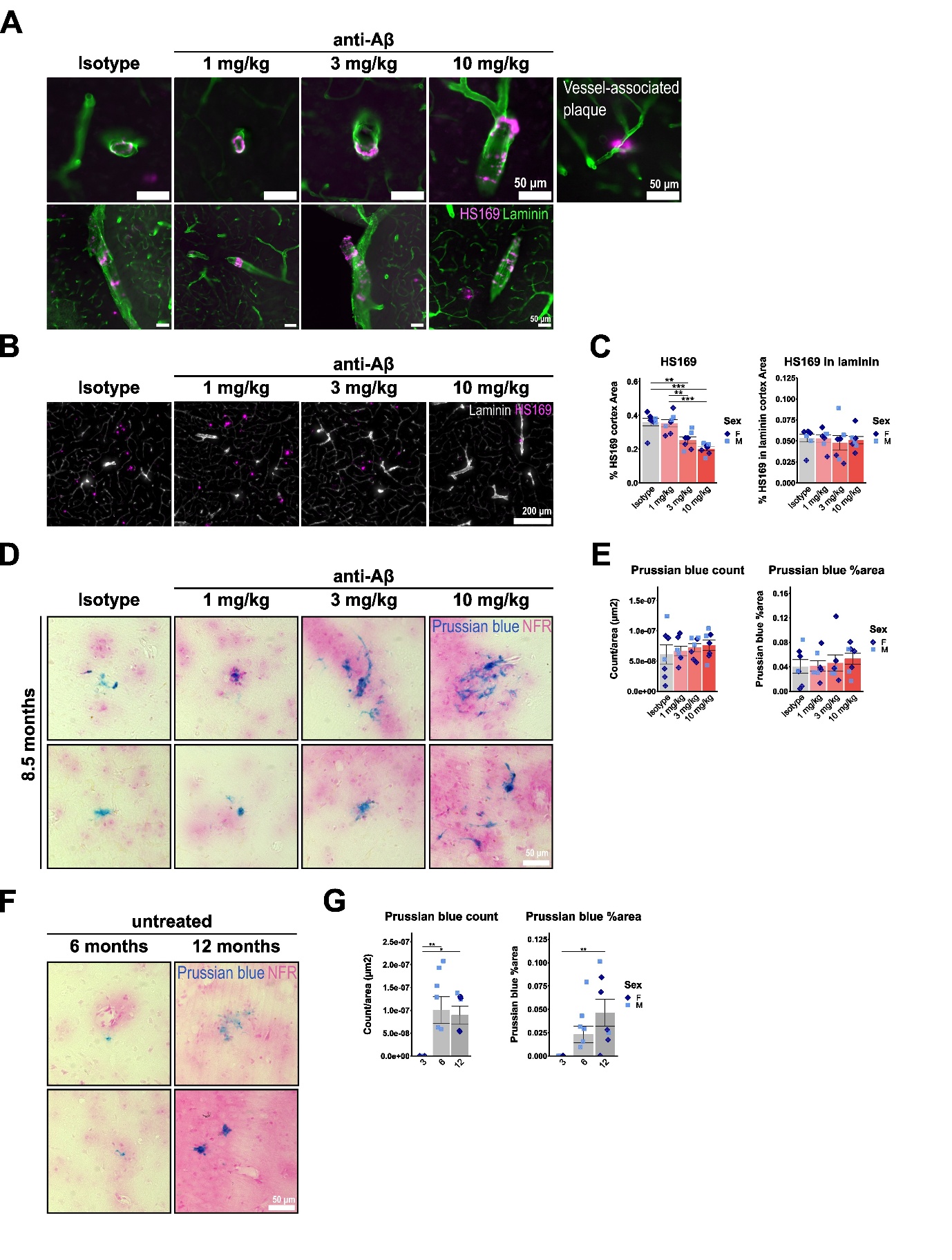
**

**Figure S3: APP-SAA KI mice develop CAA and spontaneous microhaemorrhages, which are not increased by chronic anti-Aβ treatment**(**A**) Representative immunofluorescence images of CAA as stained with HS169 in APP-SAA KI mice. (**B**) Representative immunofluorescent images of sagittal cortical sections showing co-staining of Laminin and HS169. (**C**) Quantification of percent cortical HS169 and HS169 in Laminin area. (**D**) Representative bright-field images of Prussian blue-positive deposits and microglial cell-shaped foci in anti-Aβ or isotype-treated mice. (**E**) Quantification of mean Prussian blue foci count and percent area. (**F**) Representative images of Prussian blue-positive deposits and microglial cell-shaped foci in 6- and 12-month-old APP-SAA KI mice. (**G**) Quantification of mean Prussian blue foci count and percent area. *: P < 0.05; **: P < 0.01. One-way ANOVA with Tukey’s post hoc test (C, E, G). For (C, E): isotype *n* = 5 f, 2m, 1 mg/kg *n* = 4 f, 3 m, 3 mg/kg *n* = 4 f, 3 m, 10 mg/kg *n* = 4 f, 3 m. For (G): 3 months *n* = 4 f, 3 m, 6 months *n* = 6 m, 12 months *n* = 5 f, 2 m (same samples as Fig. S1).

**
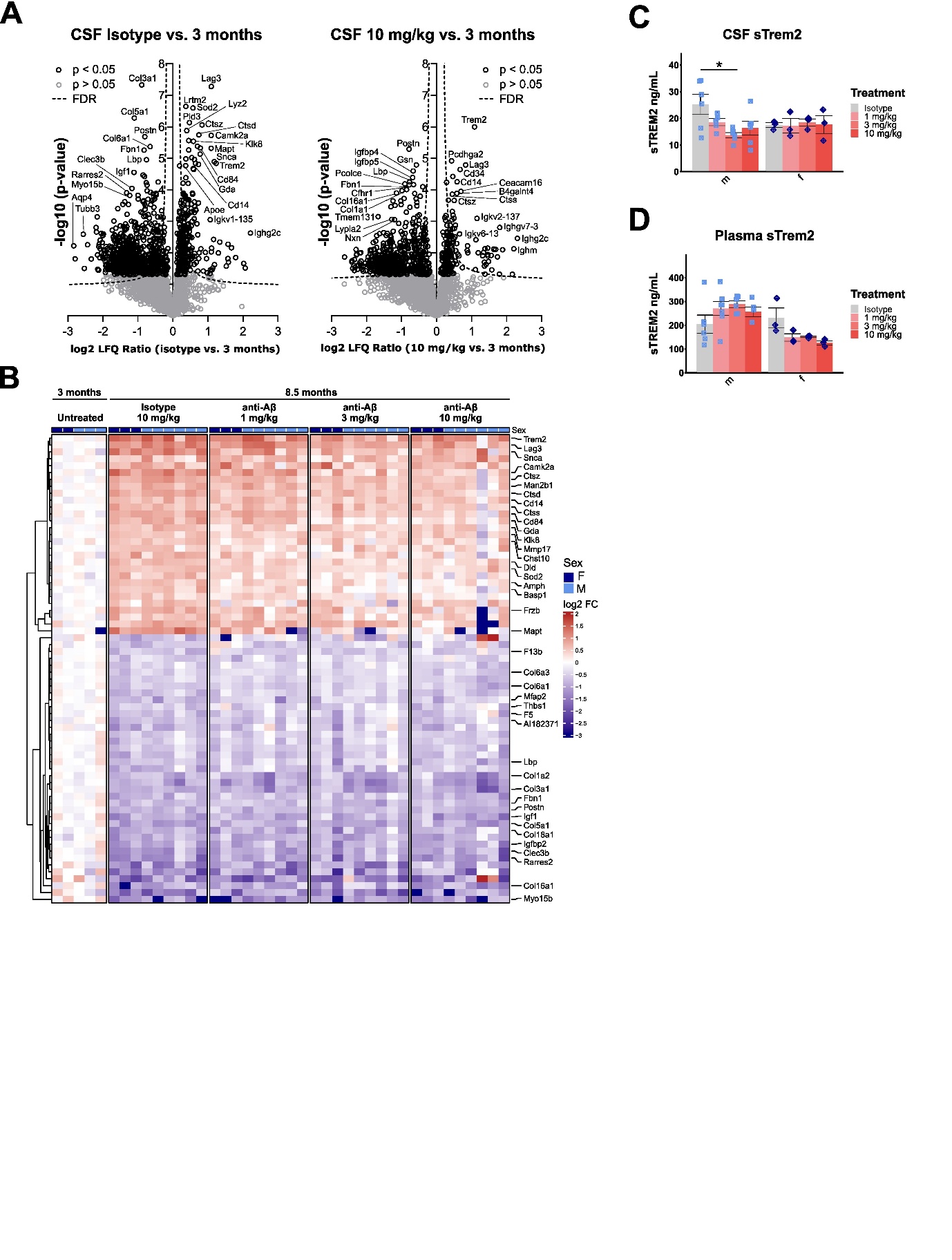
**

**Figure S4: CSF proteome changes relative to pre-disease state**
(**A**) Volcano plots showing upregulated and downregulated proteins in CSF comparing isotype or 10 mg/kg anti-Aβ-treated animals vs. 3-month-old untreated controls. (**B**) Heatmap showing significantly up- or downregulated proteins in isotype-treated 8.5-month-old mice relative to 3-month-old untreated mice (cut-off p<0.05, log_2_FC < -0.5 or >0.5). (**C**) ELISA quantification of terminally-collected CSF Trem2. (**D**) ELISA quantification of terminally-collected plasma Trem2. *: P < 0.05. Two-way ANOVA with Tukey’s post hoc test (C, D). For (A, B): untreated *n* = 2 f, 3 m, isotype *n* = 3 f, 6 m, 1 mg/kg *n* = 3 f, 6 m, 3 mg/kg *n* = 3 f, 6 m, 10 mg/kg *n* = 3 f, 6 m (same samples as Fig. 2A, B, C, Fig. S2F). For (C, D): isotype *n* = 3 f, 6 m, 1 mg/kg *n* = 3 f, 6 m, 3 mg/kg *n* = 3 f, 6 m, 10 mg/kg *n* = 3 f, 4 m (same samples as Fig. 2A, B, C, Fig. S2F).

**
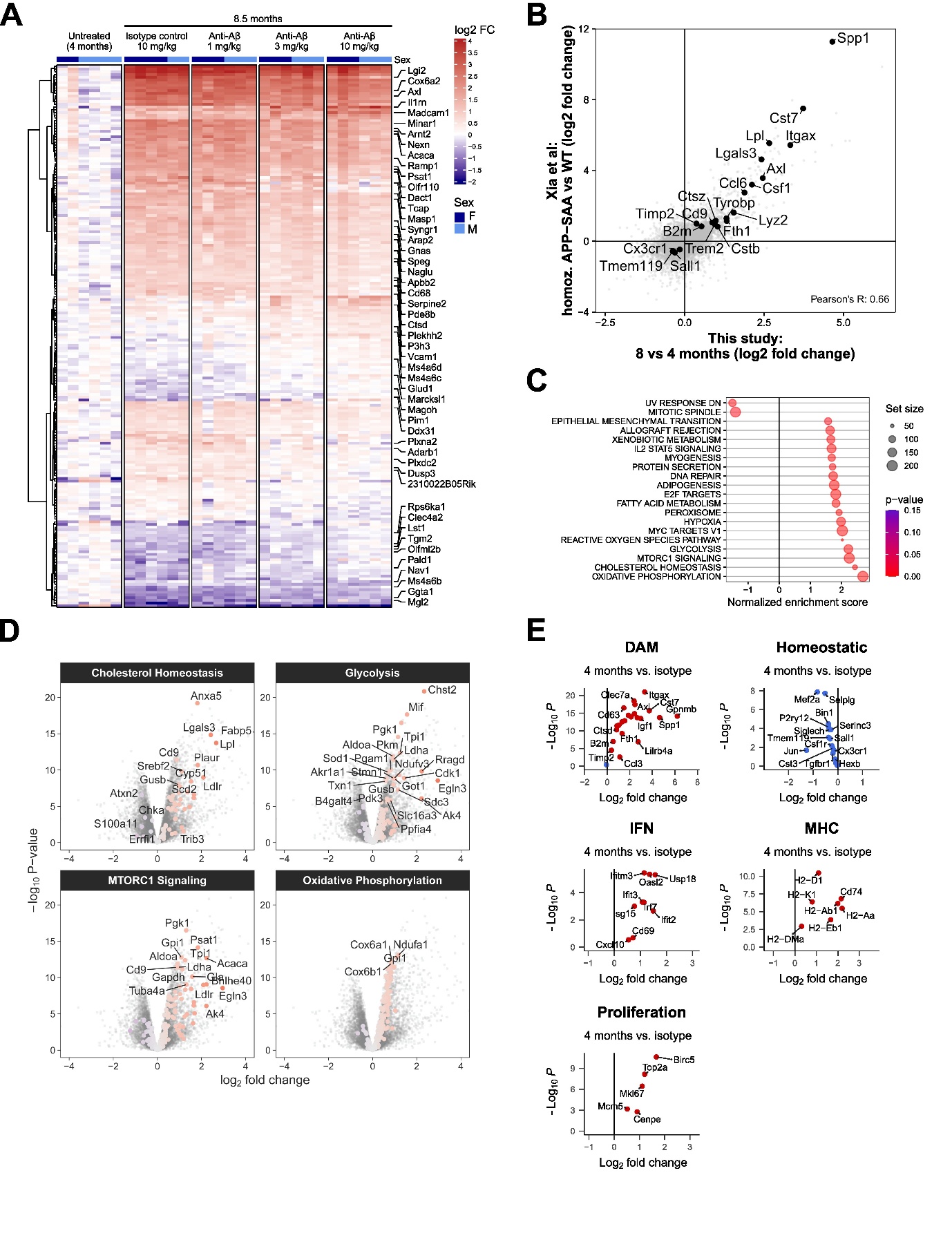
**

**Figure S5: Microglial RNA-seq gene expression changes relative to pre-disease state**(**A**) Heatmap showing differentially expressed genes relative to 4-month-old untreated mice. (**B**) Correlation plot of p-values comparing gene expression changes in isolated microglia between WT vs. 8-month-old APP-SAA KI mice from Xia et. al 2022 and changes in this study between 4-month-old untreated vs. 8-month-old isotype-treated APP-SAA KI mice. (**C**) Gene set enrichment analysis (GSEA). (**D**) Volcano plots showing differentially expressed genes related to GSEA pathways. (**E**) Volcano plots showing differentially expressed genes related to microglial states from Chen and Colonna 2021. For (A, B, C, D, E): untreated *n* = 2 f, 4 m, isotype *n* = 4 f, 2 m, 1 mg/kg *n* = 3 f, 3 m, 3 mg/kg *n* = 3 f, 3 m, 10 mg/kg *n* = 3 f, 3 m (same samples as Fig. 3A, B, C, D, F, G).

**
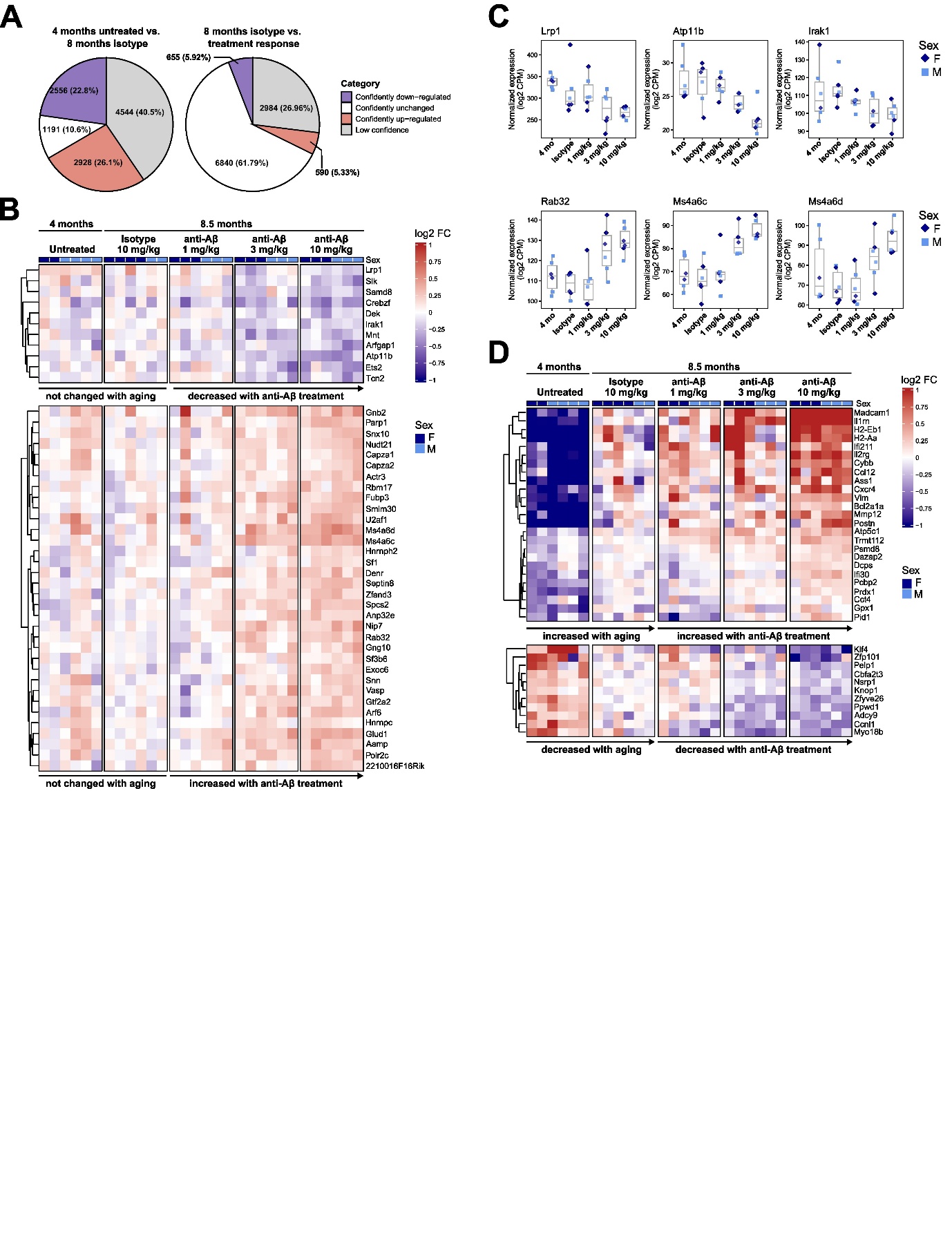
**

Figure S6: Identification of an anti-Aβ specific transcription signature

(**A**) Pie charts showing *n*-number and percentage of genes classified as “Confidently down-regulated” (purple), “Confidently unchanged” (white), “Confidently up-regulated” (pink), “Low confidence” (grey). (**B**) Heatmap showing 45 genes that are confidently unchanged with aging and confidently changed with anti-Aβ treatment. (**C**) Boxplots showing examples of genes that increase or decrease expression upon anti-Aβ treatment, but not with age. (**D**) Heatmap showing 36 genes that increase or decrease expression with age and further increase or decrease expression upon anti-Aβ treatment. Untreated *n* = 2 f, 4 m, isotype *n* = 4 f, 2 m, 1 mg/kg *n* = 3 f, 3 m, 3 mg/kg *n* = 3 f, 3 m, 10 mg/kg *n* = 3 f, 3 m.

**
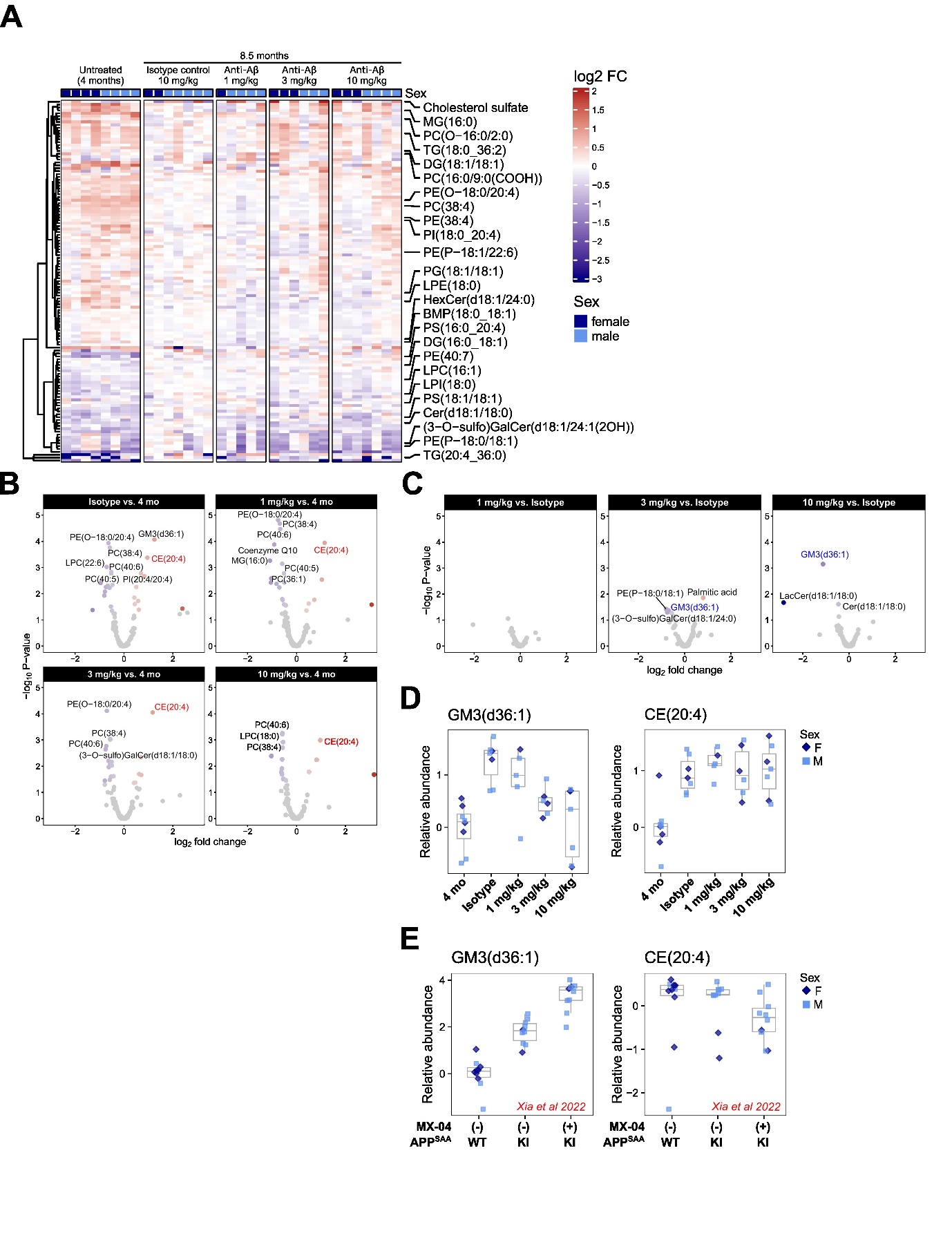
**

**Figure S7: Chronic anti-Aβ treatment does not induce lipid changes in microglia**

(**A**) Heatmap showing differentially regulated lipids relative to isotype control-treated mice. (**B**) Volcano plots showing lipid changes in isolated microglia comparing 8-month-old treated mice vs. 4-month-old APP-SAA KI mice (**C**) Volcano plots showing lipid changes in isolated microglia comparing 8-month-old treated mice vs. isotype-treated APP-SAA KI mice (**D**) Boxplots showing relative abundance of selected lipids (GM3 and CE). (**E**) Boxplots showing relative abundance of GM3 and CE from the dataset of Xia *et al.* 2022, who analysed sorted microglia from 8-month-old WT and APP-SAA mice sorted by MX-04^-^ or MX-04^+^. For (A, B, C, D): untreated *n* = 4 f, 4 m, isotype *n* = 2 f, 5 m, 1 mg/kg *n* = 1 f, 4 m, 3 mg/kg *n* = 3 f, 3 m, 10 mg/kg *n* = 3 f, 4 m. For (E): WT, MX-04^-^ *n* = 7 f, 3 m, HOM, MX-04^-^ *n* = 8 f, 2 m, HOM, MX-04^+^ *n* = 8 f, 2 m.

**
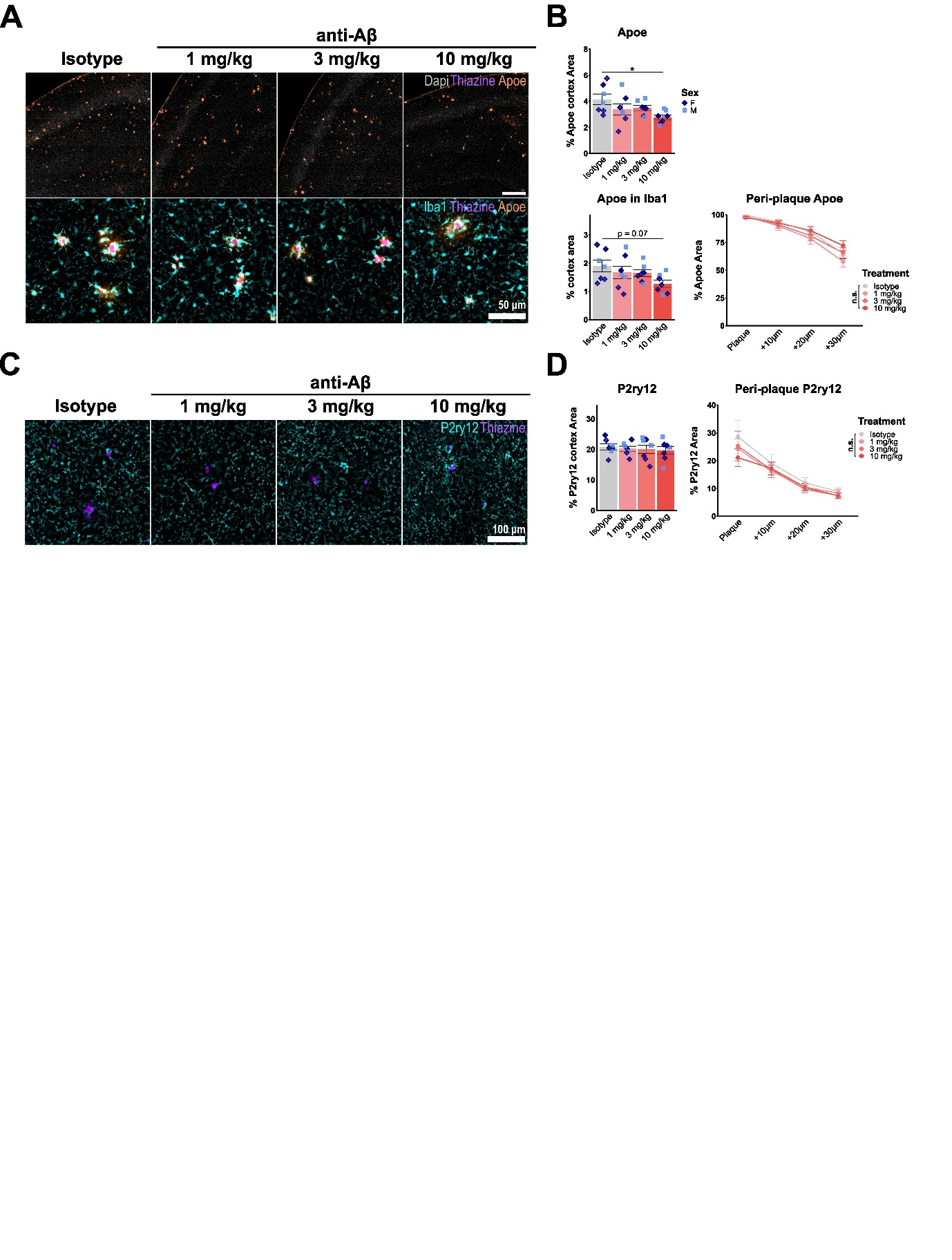
**

**Figure S8: Chronic anti-Aβ treatment decreases microglial DAM activation**(**A**) Representative epifluorescence images of DAPI (grey), Iba1 (cyan), thiazine (purple), and ApoE (orange). (**B**) Quantification of percent cortical ApoE and ApoE in Iba1, as well as concentric plaque analysis of ApoE in Iba1. (**C**) Representative epifluorescence images of P2ry12 (cyan) and thiazine (purple) (**D**) Quantification of percent cortical P2ry12, as well as concentric plaque analysis of P2ry12. *: P < 0.05, **: P < 0.01, ***: P < 0.001. One-way ANOVA with Tukey’s post hoc test (B, D). For (B, D): isotype *n* = 5 f, 2 m, 1 mg/kg *n* = 4 f, 3 m, 3 mg/kg *n* = 4 f, 3 m, 10 mg/kg *n* = 4 f, 2 m.

**
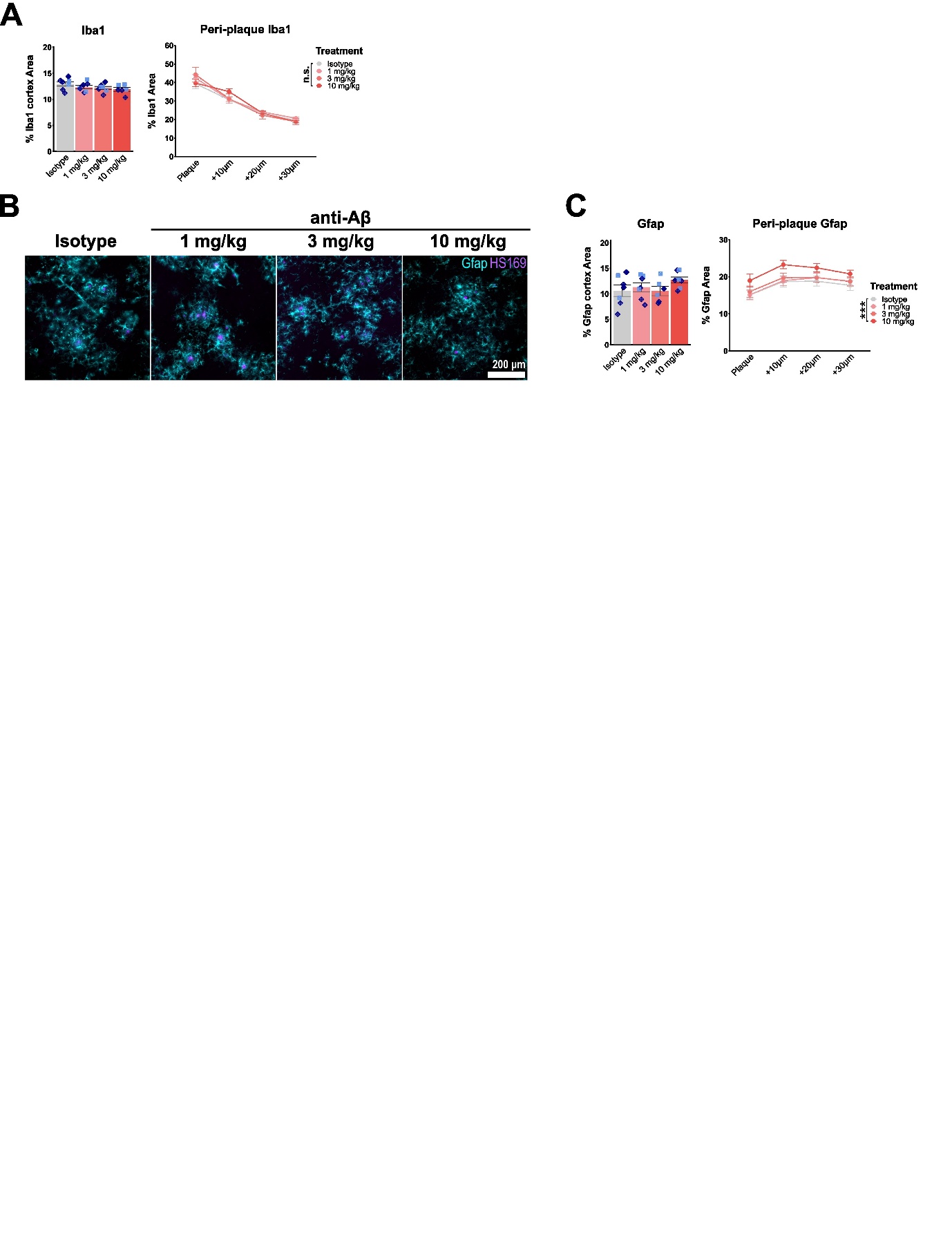
**

**Figure S9: Chronic anti-Aβ treatment does not affect microgliosis and astrogliosis, but increases Gfap around residual plaques**

(**A**) Quantification of percent cortical Iba1 and concentric-plaque analysis of Iba1. (**B**) Representative epifluorescence images of Gfap (cyan) and thiazine (purple). (**C**) Quantification of percent cortical Gfap, as well as concentric plaque analysis of Gfap. ***: P < 0.001. One-way ANOVA with Tukey’s post hoc test (A, C). For (A, C): isotype *n* = 5 f, 2 m, 1 mg/kg *n* = 4 f, 3 m, 3 mg/kg *n* = 4 f, 3 m, 10 mg/kg *n* = 4 f, 3 m.
