# Supplementary material for: Early intervention anti-Aβ immunotherapy attenuates microglial activation without inducing exhaustion at residual plaques
Source: Mol Neurodegener. 2025 Aug 20;20:92. doi: 10.1186/s13024-025-00878-1 (PMC12366171; doi:10.1186/s13024-025-00878-1)
Supplement: Supplementary file 2 — Uncropped Blots [file 13024_2025_878_MOESM2_ESM.pdf]

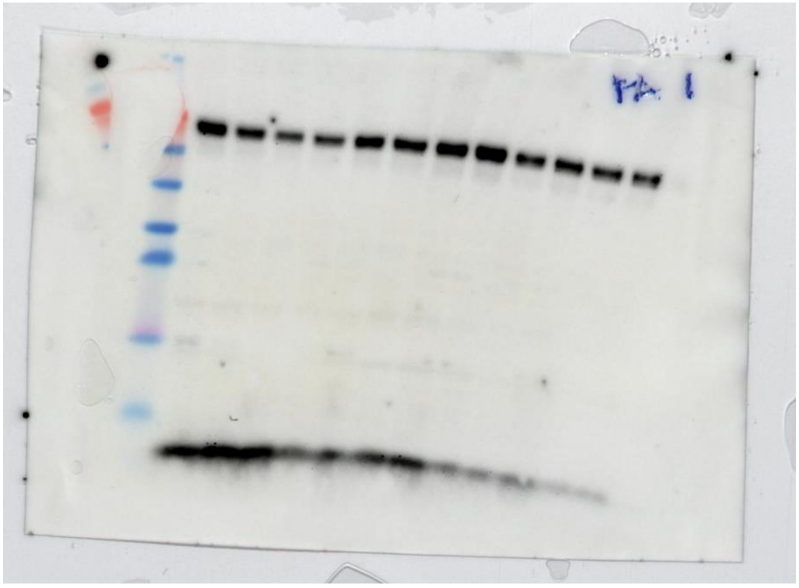

Figure 1D

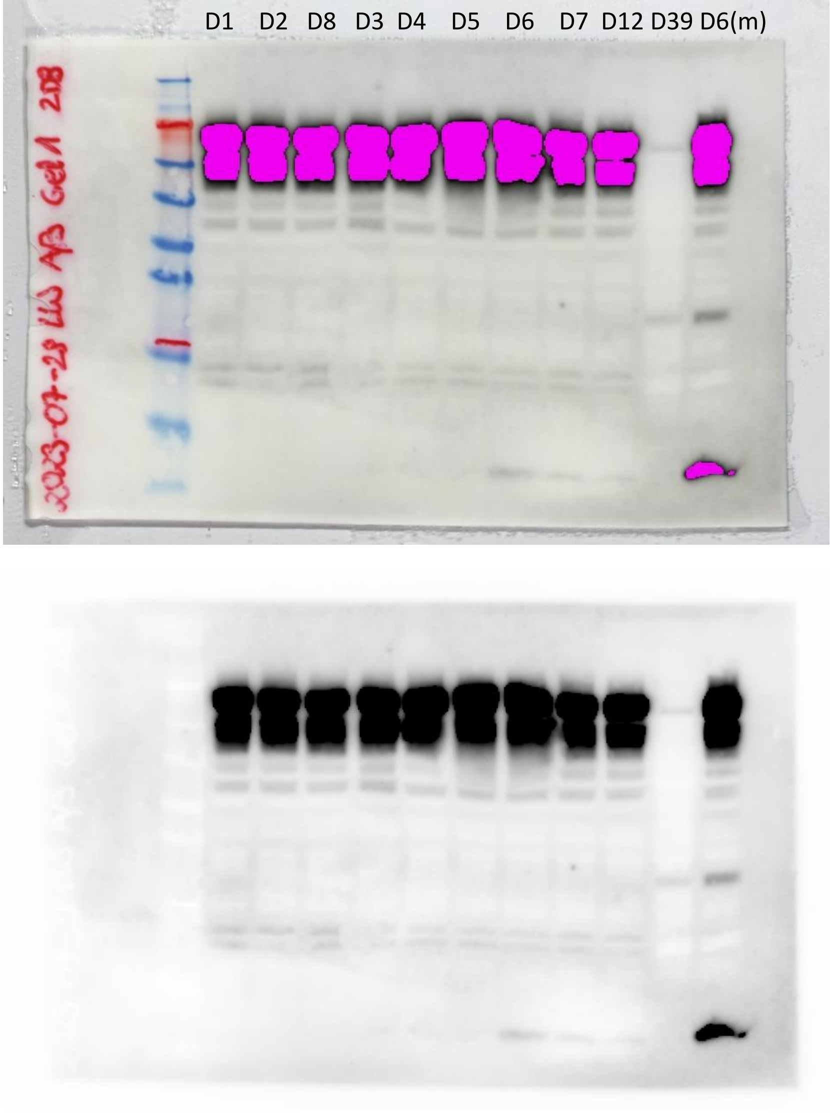

Figure S1H – DEA Samples [1, 2, 8] = 3-month-old APP-SAA, samples [3, 4, 5] = 6-month-old APP-SAA, samples [6, 7, 12] = 12-month-old APP-SAA, [39] = 14-month-old C57BL/6, [6(m)] = 18-month-old APP-SAA. D = DEA fraction

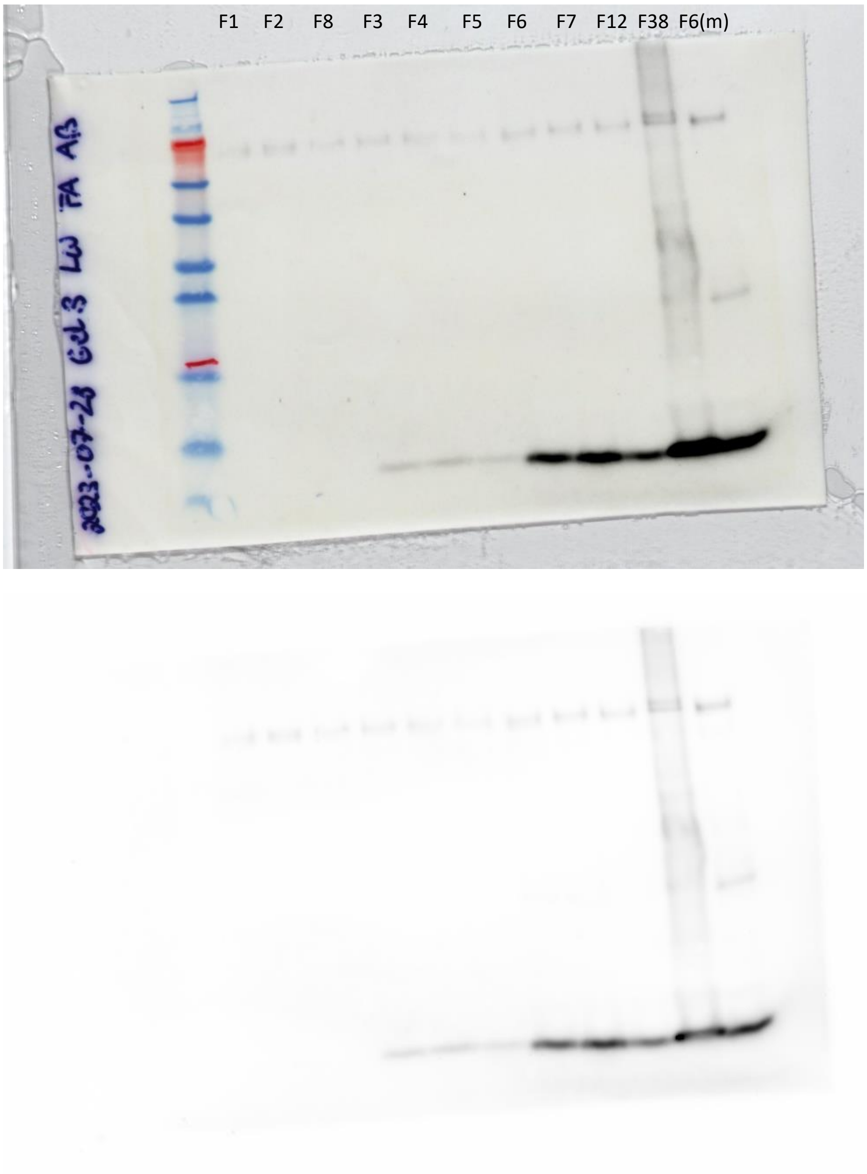

Figure S1H - FA Samples [1, 2, 8] = 3-month-old APP-SAA, samples [3, 4, 5] = 6-month-old APP-SAA, samples [6, 7, 12] = 12-month-old APP-SAA, [38] = 14-month-old APPPS1, [6(m)] = 18-month-old APP-SAA. F = FA fraction

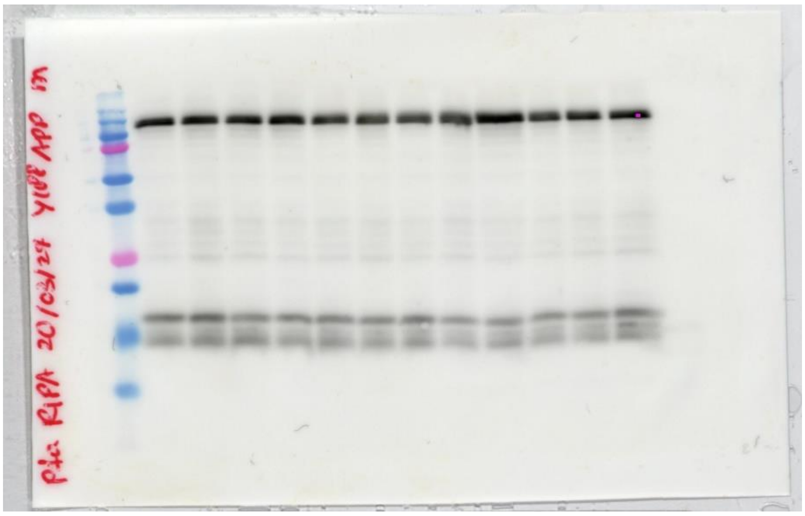

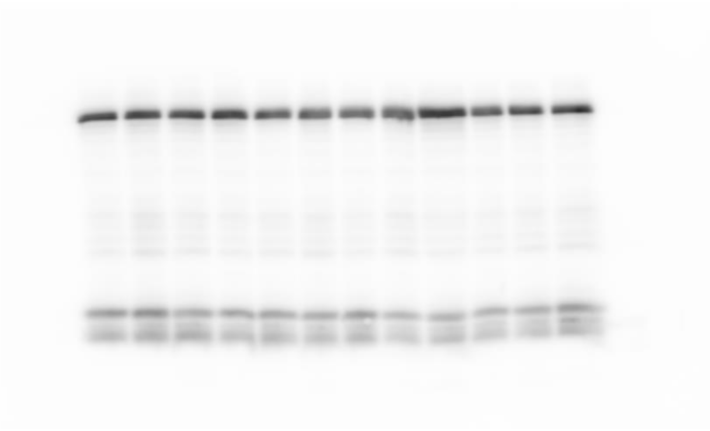

Figure S2A
