# Supplementary figures and images for: Early intervention anti-Aβ immunotherapy attenuates microglial activation without inducing exhaustion at residual plaques
Source: Mol Neurodegener. 2025 Aug 20;20:92. doi: 10.1186/s13024-025-00878-1 (PMC12366171; doi:10.1186/s13024-025-00878-1)

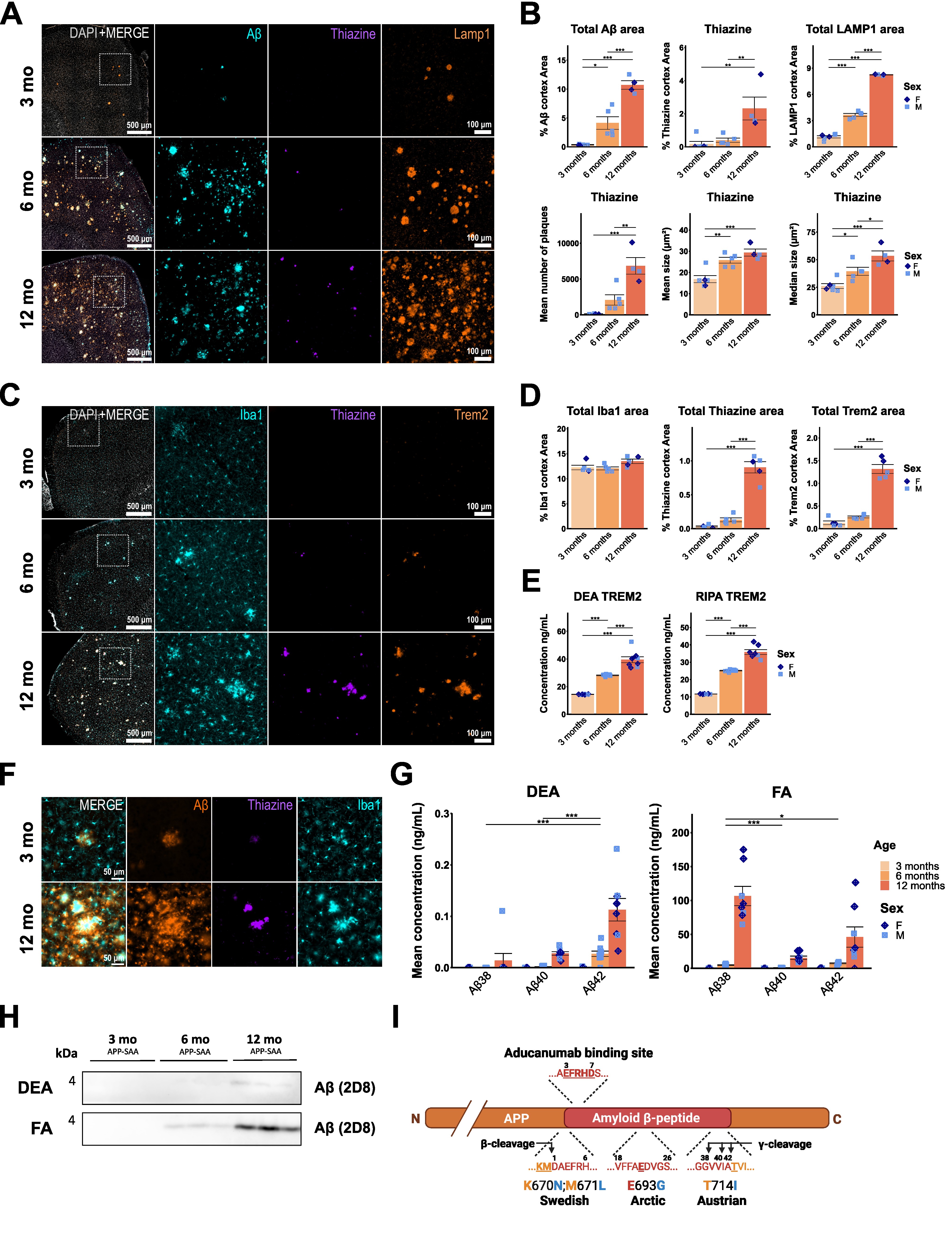

Supplement: Supplementary file 3 — Supplementary Figure 1 [file 13024_2025_878_MOESM3_ESM.jpg]

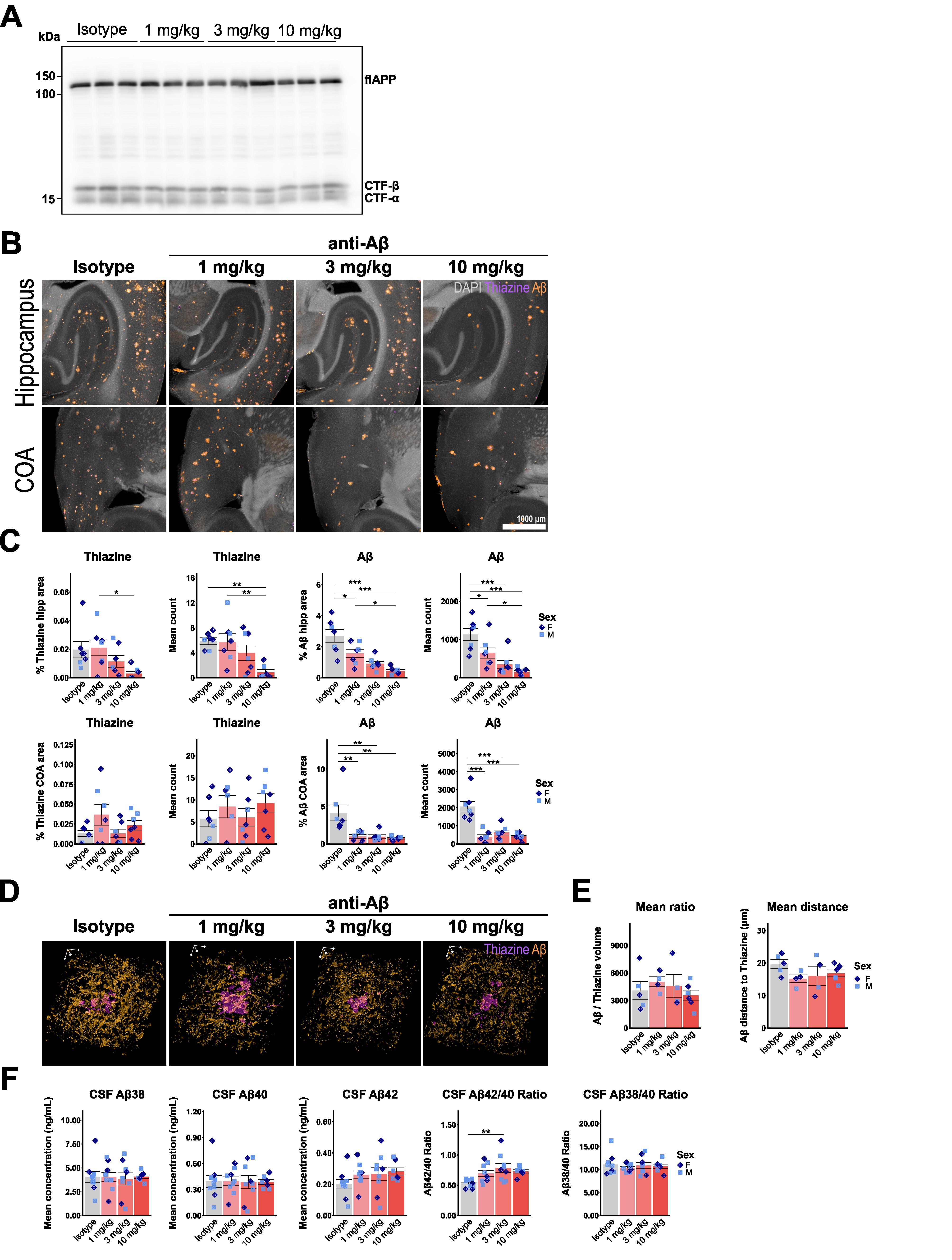

Supplement: Supplementary file 4 — Supplementary Figure 2 [file 13024_2025_878_MOESM4_ESM.jpg]

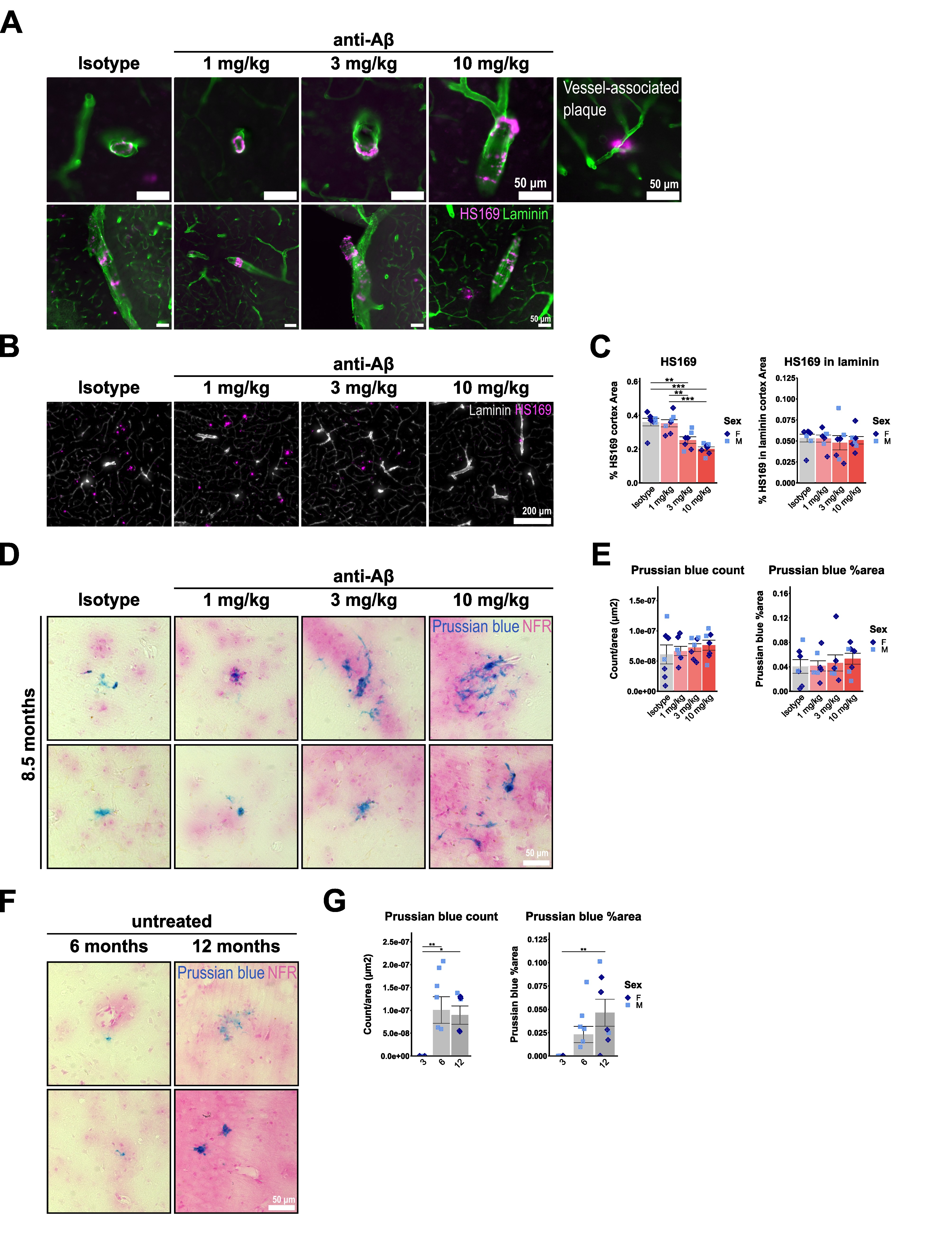

Supplement: Supplementary file 5 — Supplementary Figure 3 [file 13024_2025_878_MOESM5_ESM.jpg]

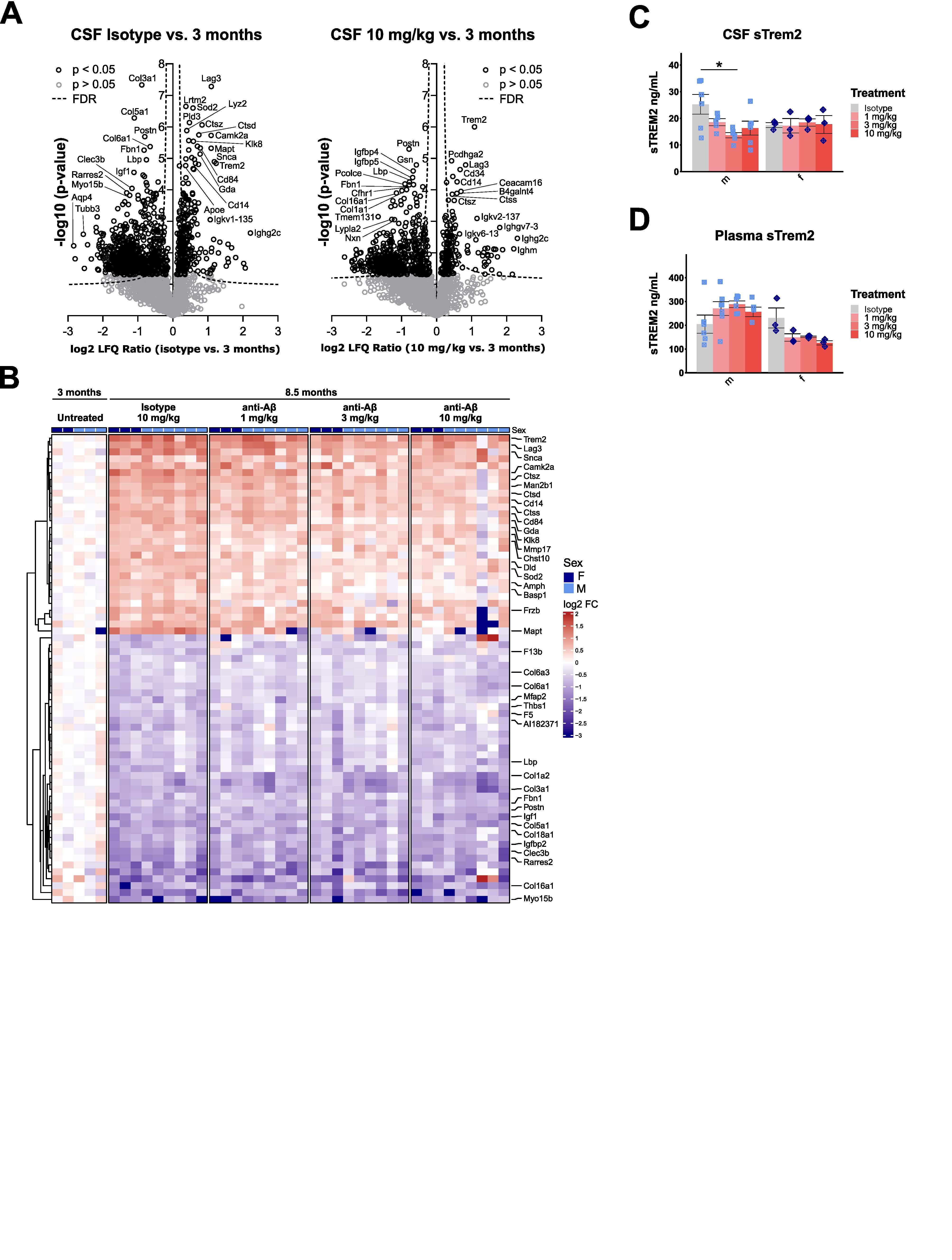

Supplement: Supplementary file 6 — Supplementary Figure 4 [file 13024_2025_878_MOESM6_ESM.jpg]

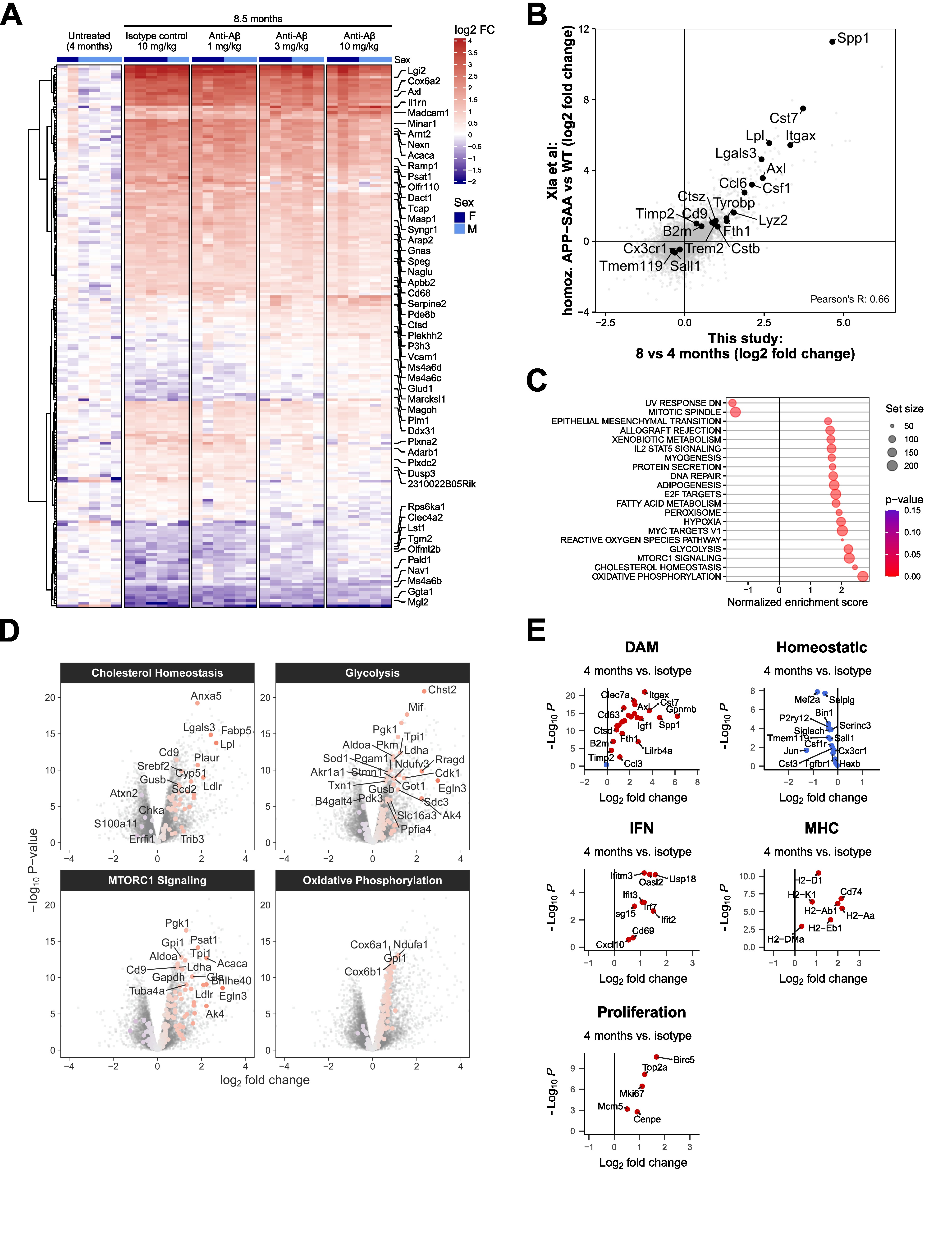

Supplement: Supplementary file 7 — Supplementary Figure 5 [file 13024_2025_878_MOESM7_ESM.jpg]

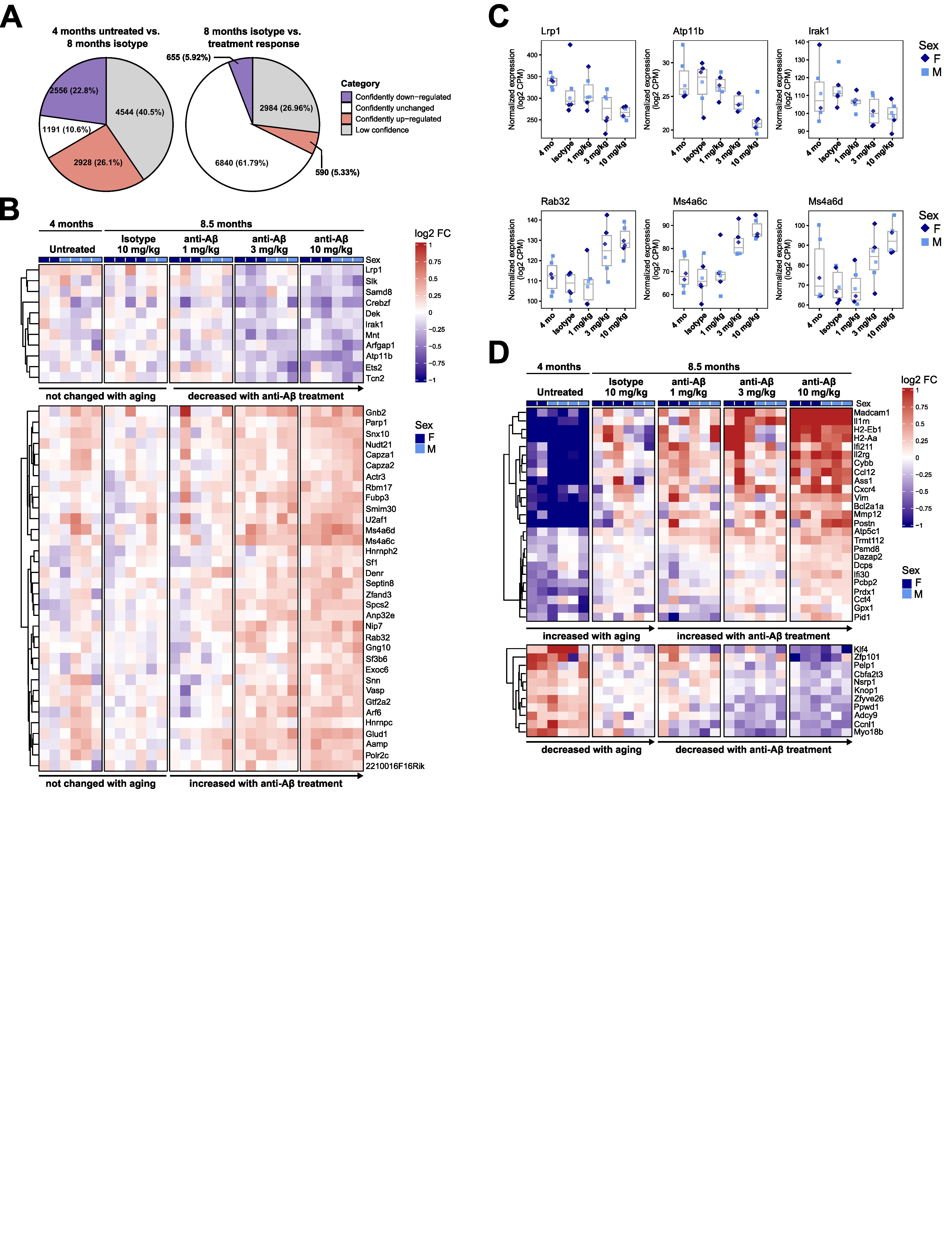

Supplement: Supplementary file 8 — Supplementary Figure 6 [file 13024_2025_878_MOESM8_ESM.jpg]

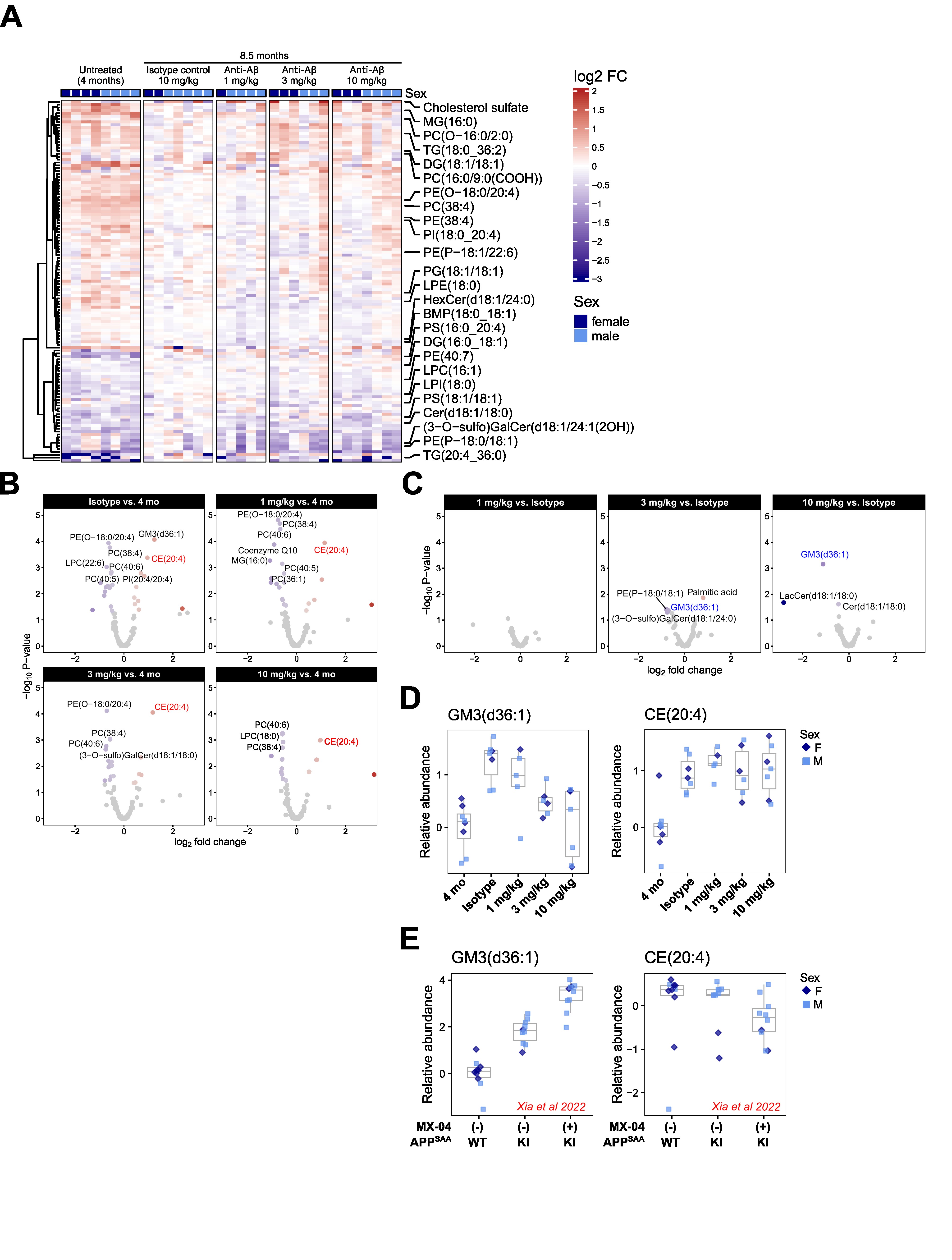

Supplement: Supplementary file 9 — Supplementary Figure 7 [file 13024_2025_878_MOESM9_ESM.jpg]

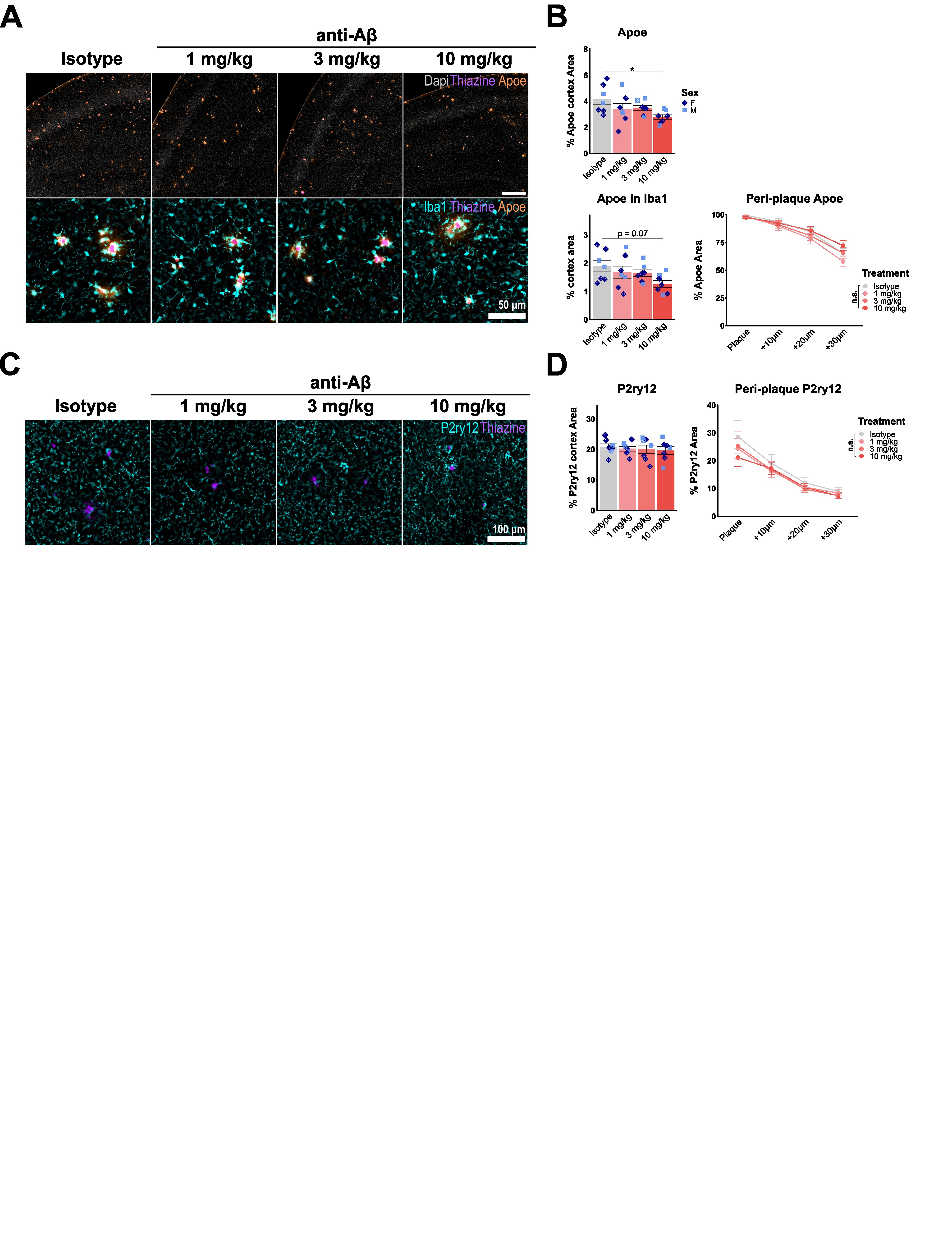

Supplement: Supplementary file 10 — Supplementary Figure 8 [file 13024_2025_878_MOESM10_ESM.jpg]

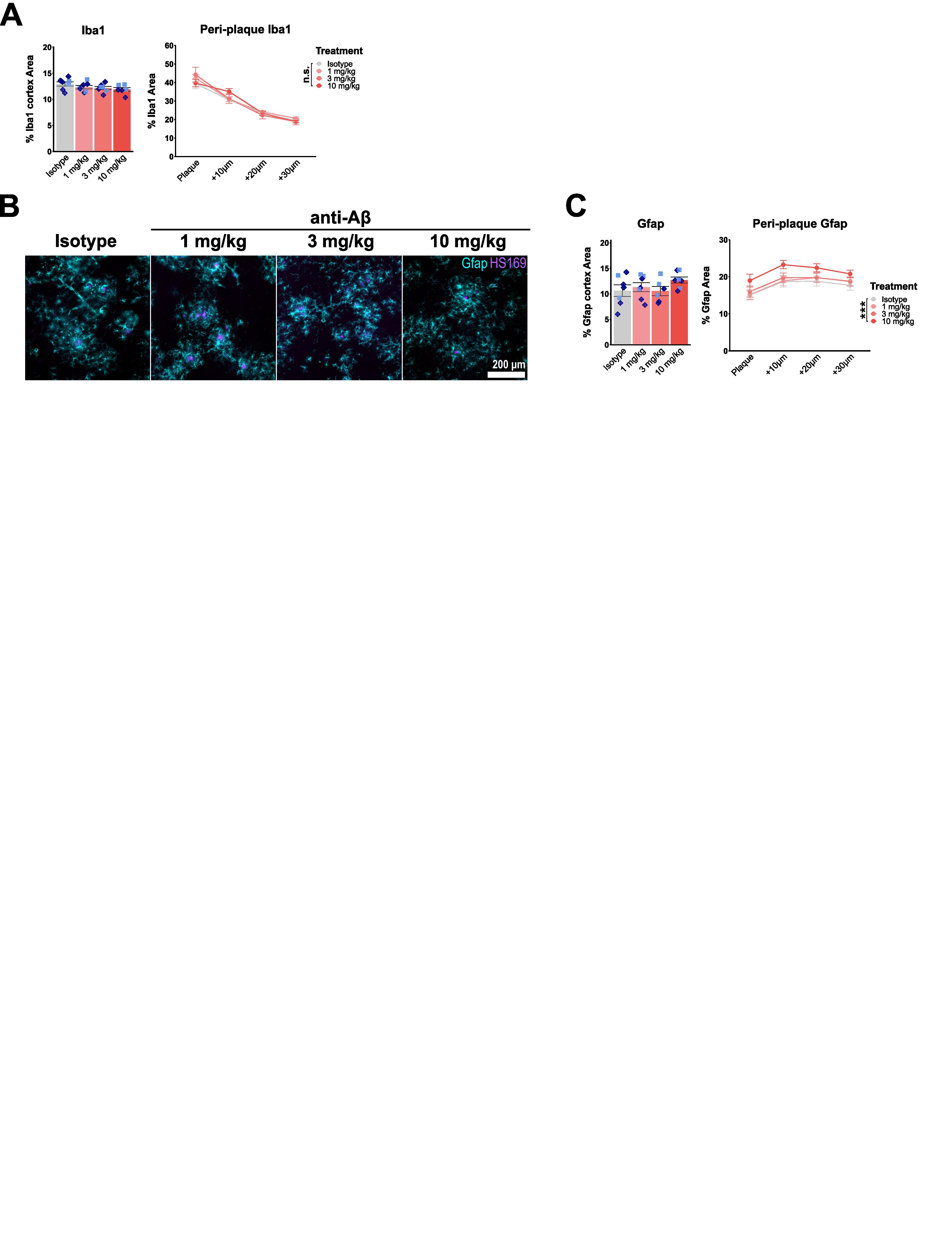

Supplement: Supplementary file 11 — Supplementary Figure 9 [file 13024_2025_878_MOESM11_ESM.jpg]
